# The HUSH complex cooperates with TRIM28 to repress young retrotransposons and new genes

**DOI:** 10.1101/gr.228171.117

**Published:** 2018-06

**Authors:** Luisa Robbez-Masson, Christopher H.C. Tie, Lucia Conde, Hale Tunbak, Connor Husovsky, Iva A. Tchasovnikarova, Richard T. Timms, Javier Herrero, Paul J. Lehner, Helen M. Rowe

**Affiliations:** 1 Infection and Immunity, University College London, London WC1E 6BT, United Kingdom;; 2 Bill Lyons Informatics Centre, UCL Cancer Institute, University College London, London WC1E 6DD, United Kingdom;; 3 Cambridge Institute for Medical Research, University of Cambridge, Cambridge CB2 0XY, United Kingdom

## Abstract

Retrotransposons encompass half of the human genome and contribute to the formation of heterochromatin, which provides nuclear structure and regulates gene expression. Here, we asked if the human silencing hub (HUSH) complex is necessary to silence retrotransposons and whether it collaborates with TRIM28 and the chromatin remodeler ATRX at specific genomic loci. We show that the HUSH complex contributes to de novo repression and DNA methylation of an SVA retrotransposon reporter. By using naïve versus primed mouse pluripotent stem cells, we reveal a critical role for the HUSH complex in naïve cells, implicating it in programming epigenetic marks in development. Although the HUSH component FAM208A binds to endogenous retroviruses (ERVs) and long interspersed element-1s (LINE-1s or L1s), it is mainly required to repress evolutionarily young L1s (mouse-specific lineages <5 million years old). TRIM28, in contrast, is necessary to repress both ERVs and young L1s. Genes co-repressed by TRIM28 and FAM208A are evolutionarily young, or exhibit tissue-specific expression, are enriched in young L1s, and display evidence for regulation through LTR promoters. Finally, we demonstrate that the HUSH complex is also required to repress L1 elements in human cells. Overall, these data indicate that the HUSH complex and TRIM28 co-repress young retrotransposons and new genes rewired by retrotransposon noncoding DNA.

Although <2% of DNA sequence in the human genome codes for proteins, the vast majority plays an enigmatic role and has thus been referred to as genomic dark matter (Diederichs et al. 2016). However, this extra DNA serves a purpose: First, it contains regulatory elements that control when and where genes are expressed, a role only now being realized (Sanjana et al. 2016). Second, it is involved in building heterochromatin, for example at the nuclear periphery (Lemaitre and Bickmore 2015). Little is understood about how heterochromatin is formed, but its content is dominated by retrotransposons, which contribute to its establishment from plants to mammals (Lippman et al. 2004; Matsui et al. 2010). Retrotransposons replicate through an RNA intermediate, which has allowed them and their regulatory sequences to accumulate and coevolve with their hosts (Robbez-Masson and Rowe 2015; Thompson et al. 2016).

The human silencing hub (HUSH) complex, composed of FAM208A (also known as TASOR), MPHOSPH8 (also known as mpp8), and PPHLN1 (periphilin 1) is recruited to genomic loci rich in H3K9me3 (Brummelkamp and van Steensel 2015; Tchasovnikarova et al. 2015; Timms et al. 2016) and interacts with SETDB1 and MORC2 (Tchasovnikarova et al. 2017). The HUSH complex mediates position-effect variegation at reporter constructs that are integrated into silent chromatin (Tchasovnikarova et al. 2015), and depletion of HUSH components reduces H3K9me3 and alters transcription (Timms et al. 2016). It is unknown whether the HUSH complex is required for the repression of retrotransposons.

TRIM28, in contrast, is known to silence retrotransposons early in development (Rowe et al. 2010; Turelli et al. 2014) and is targeted to DNA through KRAB-zinc finger proteins (KZFPs in mouse or KZNFs in human), most of which are specific for transposon sequences (Wolf and Goff 2009; Jacobs et al. 2014; Schmitges et al. 2016; Imbeault et al. 2017). TRIM28 recruits chromatin writers, readers, and erasers including SETDB1, CBX5 (Hp1alpha), and CHAF1A (Lechner et al. 2000; Ivanov et al. 2007; Matsui et al. 2010; Yang et al. 2015). The resulting silent H3K9me3 mark at retrotransposons overlaps with H3F3A/B (histone variant 3.3), ATRX, and DAXX (Elsasser et al. 2015; He et al. 2015; Sadic et al. 2015; Wolf et al. 2017) and spreads to nearby genes (Karimi et al. 2011; Rebollo et al. 2011; Rowe et al. 2013b; Hummel et al. 2017).

We asked here if the HUSH complex, like TRIM28, is necessary for retrotransposon repression and whether it cooperates with TRIM28 and ATRX at specific genomic loci.

## Results

### The HUSH complex contributes to repression of an SVA retrotransposon reporter

**Figure 1.**
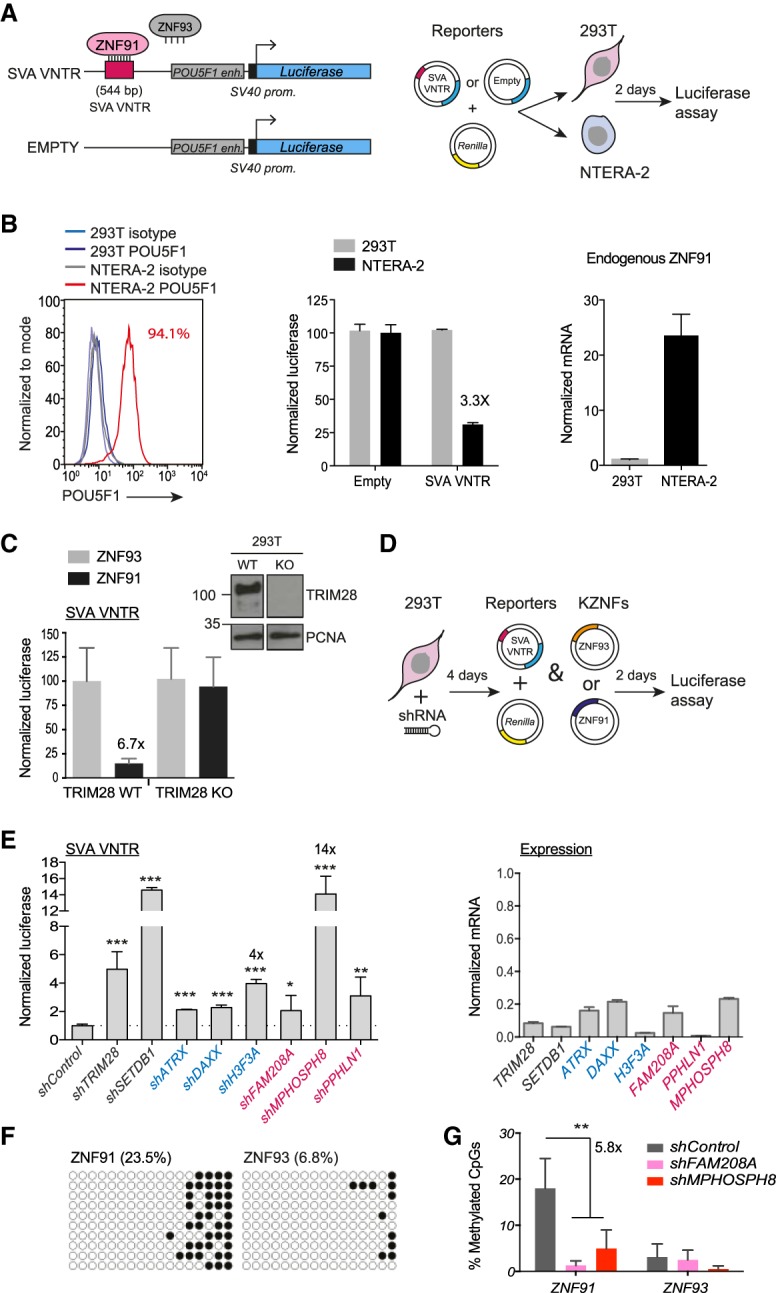
The HUSH complex contributes to repression and DNA methylation of an SVA retrotransposon reporter. (*A*, *left*) ZNF91 binds the SVA VNTR sequence and represses the reporter. ZNF93 or an empty reporter were used as controls. (*enh*.) enhancer; (*prom*.) promoter; (SV40) simian virus 40. (*Right*) Either luciferase reporter was cotransfected with a *Renilla* luciferase-encoding control, and relative luciferase light units were measured 48 h later. (*B*) NTERA-2 cells were verified to express POU5F1 (*left*) and subject to reporter assays shown in *A* (*middle*), here normalized to the empty control, and *ZNF* expression was measured by qRT-PCR (*right*). (*C*) Reporter assays in *TRIM28* WT and KO 293T cells, including cotransfection of stated exogenous ZNFs. Data are normalized to the bar on the *left* (*TRIM28* WT, *ZNF93*). Western blot for TRIM28 using PCNA as a loading control (*insets* from the same blot). (*D*) 293T cells were transduced with shRNA vectors against epigenetic factors and puromycin-selected before reporter assays. (*E*) Following the assay in *D*, data for *ZNF91* were normalized to *ZNF93* (*left*). Unpaired *t*-tests were used to assess differences between the *shControl* (transduced with the same vector lacking a hairpin that was puromycin-selected in parallel) and the target shRNA. Experiments were repeated at least three times for each candidate, and representative data are shown. Two-tailed unpaired *t*-tests were done: (*) *P* = <0.05; (**) *P* = <0.01; (***) *P* = <0.001. qRT-PCR was used to assess the knockdown efficiency of each mRNA (*right*). (*F*) DNA methylation analysis of the SV40 promoter 48 h post reporter transfection. Plasmids were produced in dam^−^ bacteria. Methylated and unmethylated CpGs are shown as filled and open circles, respectively. (*G*) Summary results of levels of de novo DNA methylation of the reporter in control cells versus HUSH-depleted cells. See also Supplemental Figure S1G. Two-tailed, unpaired *t*-tests were used to compare four controls to four HUSH-depleted samples (from two independent experiments): *P* = 0.0062.

We used a human retrotransposon reporter (Fig. 1A) that is repressed through ZNF91 binding to an SVA-type D variable number tandem repeat (SVA VNTR) sequence (Jacobs et al. 2014). We found that the SVA reporter was repressed (3.3×) in POU5F1-positive human embryonal NTERA-2 cells, which naturally express *ZNF91*, but not in 293T cells (Fig. 1B). Reporter repression could be engineered in POU5F1-expressing mouse embryonic stem cells (mESCs) by transfection of the cognate *ZNF91*, but not of a control KZNF, *ZNF93* as expected (Supplemental Fig. S1A; Jacobs et al. 2014), and even in 293T cells (Supplemental Fig. S1B). We used 293T cells for subsequent reporter assays because of their amenity to genetic manipulation.

We validated that reporter repression (6.7×) (Fig. 1C) was TRIM28-dependent by using *TRIM28* knockout and complemented 293T cells. Having set up this system, we depleted the HUSH complex using shRNA before introduction of the reporter gene (Fig. 1D). All three HUSH components contributed to SVA repression (Fig. 1E), with the most striking effect observed for *MPHOSPH8* depletion (14× derepression). Although these results were obtained with single hairpins, we validated the role of *Mphosph8* with an independent hairpin in mESCs (Supplemental Fig. S1C). Considering that the HUSH complex is thought to function similarly to CBX proteins (Brummelkamp and van Steensel 2015), we depleted all three CBX family members, which revealed that they too all contributed to repression in 293Ts and HeLa cells (Supplemental Fig. S1D). Finally, we identified a role for H3F3A (4× derepression) and its ATRX–DAXX chaperone complex in establishing SVA repression (Fig. 1E; Supplemental Fig. S1C).

### De novo DNA methylation of the SVA reporter depends on the HUSH complex

We reasoned that the HUSH complex may be targeted to the repressed SVA reporter through the chromodomain of MPHOSPH8 that interacts with H3K9me3 (Kokura et al. 2010); indeed, we found H3K9me3 to be enriched (2.4×) on the repressed reporter (Supplemental Fig. S1E). Of note, plasmids are chromatinized and subject to H3K9me3 (Barde et al. 2009). We also detected cognate ZNF-specific de novo DNA methylation (23.5%) (Fig. 1F), as observed before for retroviral reporters (Wolf and Goff 2009; Rowe et al. 2013a). DNA methylation was not necessary for repression but contributed to it, since we detected 4.5× SVA repression in *Dnmt* knockout mESCs, compared to 9× repression in wild-type mESCs (Supplemental Fig. S1F). This suggested HUSH may be necessary for handover of H3K9me3 to DNA methylation and in support of this, depletion of HUSH components coincided with a decrease (5.8×) in de novo DNA methylation at the SV40 promoter along with a reduction (up to 2.7×) in SVA reporter repression (Fig. 1G; Supplemental Fig. S1G). In sum, these data on the nonintegrated reporter suggest that HUSH may be required for the maintenance of retrotransposon repression.

### The HUSH complex is critical for endogenous retrotransposon repression in naïve pluripotent cells

**Figure 2.**
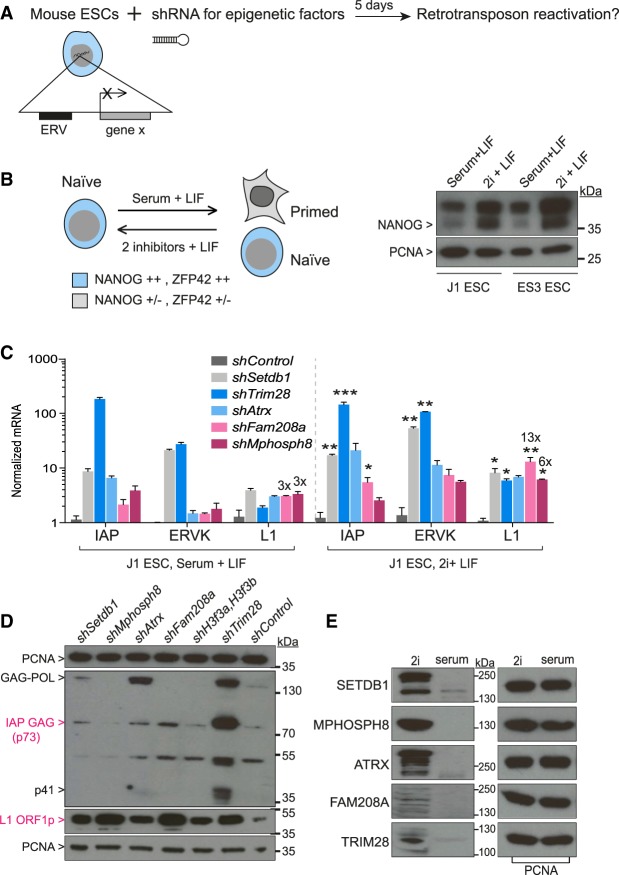
The HUSH complex is critical for endogenous retrotransposon repression in naïve pluripotent cells. (*A*) Endogenous retrotransposon expression was measured by qRT-PCR following shRNA-depletion of epigenetic modifiers in mESCs. (*B*) Naïve cells express higher levels (++) of ZFP42 (REX1) and NANOG (*left*), the latter shown by Western blot in two mESC strains (*right*). Predicted band sizes: NANOG, 34 kDa; PCNA, 29 kDa. (*C*) Endogenous retrotransposon expression following depletion of epigenetic modifiers. One representative experiment of three is shown. *Atrx* was not examined in the third experiment, excluding it from statistical analyses. Two-tailed paired *t*-tests were done for 2i + LIF samples. (*D*) Western blot for IAP GAG p73 using a rabbit IAP GAG antibody or PCNA as control in 2i + LIF J1 ESCs. The antibody detects p73 as well as GAG-POL and GAG cleavage products, including p41, representing partially processed GAG. Samples were re-run on a second gel and reblotted for L1 ORF1 protein (40 kDa) and reprobed for PCNA. (*E*) J1 ESCS grown in serum versus 2i conditions were blotted for epigenetic factors or PCNA as a normalizer. Predicted band sizes: SETDB1, 143 kDa; MPHOSPH8, 97 kDa; ATRX, 280 kDa; FAM208A, 200 kDa; KAP1, 100 kDa.

We reasoned that the HUSH complex may exert its greatest impact in naïve pluripotent cells, in which chromatin awaits stable epigenetic programming (Ying et al. 2008; Ficz et al. 2013). We depleted epigenetic factors in mESCs (Fig. 2A) and compared mixed population to naïve cultures, the latter in which we verified enhanced NANOG expression (Fig. 2B). Depletion of *Mphosph8*, as well as *Atrx* and *Trim28*, was sufficient to reactivate retrotransposons in two different strains of serum-cultured mESCs (Supplemental Fig. S2A). However, although *Trim28* or *Atrx* depletion mainly affected intracisternal A-particle (IAP) elements, *Mphosph8* depletion mainly affected L1s. Parallel culture of J1 ESCs in serum versus 2i conditions consistently led to more pronounced reactivation in the naïve cells (Fig. 2C; Supplemental Fig. S2B) with HUSH components *Mphosph8* and *Fam208a* both affecting L1 elements (up to 13× reactivation in naïve cells) (Fig. 2C). Depletion of *Trim28*, *Setdb1*, *Atrx*, or *Fam208a* resulted in IAP GAG protein accumulation, whereas depletion of *Fam208a* and *Mphosph8* led to increased production of L1 ORF1 protein (Fig. 2D). Finally, TRIM28, SETDB1, ATRX, MPHOSPH8, and FAM208A protein expression levels were elevated in naïve cells (Fig. 2E), in support of their critical role early in development (Cammas et al. 2000; Dodge et al. 2004; Garrick et al. 2006; Harten et al. 2014).

### TRIM28 and FAM208A co-repress a set of protein-coding genes

**Figure 3.**
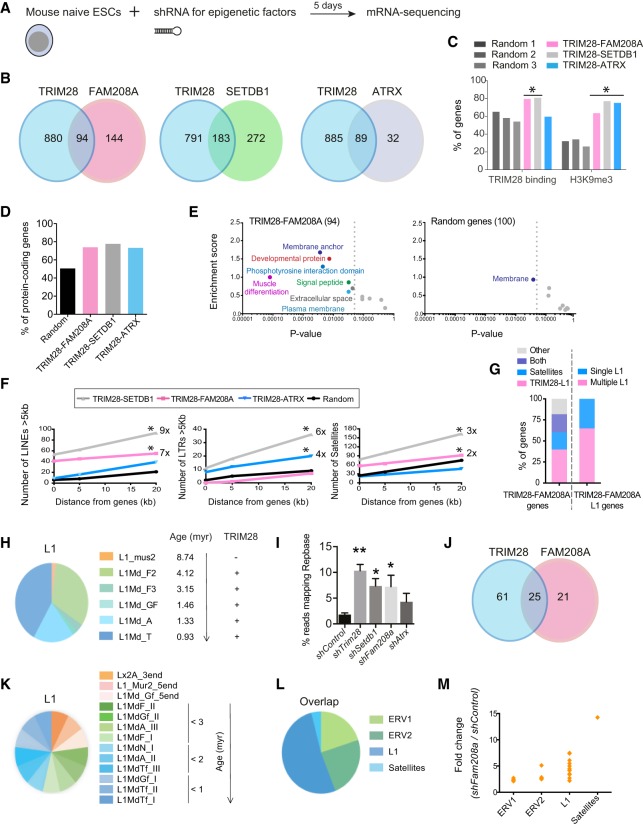
TRIM28 and FAM208A exert nonredundant roles at evolutionarily young L1s and associated genes. (*A*) Naïve knockdown J1 mESCs were subject to mRNA-sequencing. Biological replicates were sequenced from three independent experiments. (*B*) Genes up-regulated >2× (where *P*_adj_ ≤ 0.05) in each treatment group showing the overlap between groups. (*C*) The three gene sets or three random gene sets (the latter containing 100 per group) were examined for the presence of a TRIM28 or H3K9me3 peak within a radius of 20 kb. (*D*) Percentage of protein-coding genes in each group. (*E*) Gene ontology (DAVID analysis) of the 94 TRIM28-FAM208A repressed genes (*left*, seven gene clusters were enriched with *P*-values <0.05) and the 100 random genes (*right*). (*F*) UCSC Table Browser analysis showing the number of the stated repeats located within increasing distances (0, 5, and 20 kb) of the sets of genes. Significant gene sets are marked and the fold change relative to random genes at intersection (0 kb) is stated where different. (*Left*) TRIM28-FAM208A, *P* = 0.000025; TRIM28-SETDB1, *P* = 0.002540; (*middle*) TRIM28-ATRX, *P* = 0.010200; TRIM28-SETDB1, *P* = 0.035700; (*right*) TRIM28-FAM208A, *P* = 0.00643; TRIM28-SETDB1, *P* = 0.00428. (*G*) The percentage of TRIM28-FAM208A genes that contain the stated repeats (*left* bar) or the percentage of L1-containing TRIM28-FAM208A genes that contain multiple L1s (*right* bar). Only TRIM28-dependent L1s are considered (from the families L1Md_F, L1Md_F2, L1Md_F3, L1Md_A, and L1Md_T). (*H*) Full-length (>5 kb) L1 elements located within 20 kb of the TRIM28-FAM208A genes were classified according to family and mean age of that family and whether (+/−) they bind TRIM28. (*I*) The percentage of reads mapping Repbase within each treatment group is shown (*n* = 3, except for ATRX where *n* = 2). Error bars represent standard deviation or standard error (ATRX). (*J*) Venn diagram showing 25 repeat families are co-repressed by TRIM28 and FAM208A. They are defined as >2× up-regulated (*P* = <0.05) in both *Trim28* and *Fam208a*-depleted cells. (*K*) All L1 families co-repressed by TRIM28 and FAM208A are classified here by name and age. (*L*) Proportion of repeats from each class that are co-repressed by TRIM28 and FAM208A. (*M*) The same repeat families as *L*, but here, their up-regulation in *Fam208a*-depleted cells is shown.

We sought to identify genomic sites where TRIM28 collaborates with FAM208A, SETDB1, and ATRX and therefore performed mRNA-sequencing (Fig. 3A) and focused on up-regulated genes. Samples within treatment groups clustered together (Supplemental Fig. S3A) and a large proportion of TRIM28-repressed genes were co-repressed by FAM208A (94 genes), SETDB1 (183 genes), or ATRX (89 genes) (Fig. 3B; Supplemental Fig. S3B; Supplemental Table S4). These three groups of TRIM28-repressed genes are likely direct rather than indirect targets, because up to 81% overlapped TRIM28 peaks (TRIM28-FAM208A and TRIM28-SETDB1 gene sets) and up to 77% overlapped H3K9me3 (all three gene sets), compared to randomly-selected gene groups (“Random”) (Fig. 3C). All three groups were enriched in protein-coding genes (Fig. 3D), and follow-up of the TRIM28-FAM208A genes revealed them to be functionally related and involved in developmental pathways (Fig. 3E), unlike random genes. Of note, we verified that up-regulation was detectable across the length of the transcripts within this group (Supplemental Fig. S3C).

### TRIM28 and FAM208A exert nonredundant roles in repressing young L1 elements

TRIM28 regulates genes through binding repeats (Rowe et al. 2013b; Hummel et al. 2017). We therefore asked if these sets of genes were enriched in repeats and divided repeats by size (Fig. 3F; Supplemental Fig. S3D). TRIM28-SETDB1 genes were enriched for all repeats here (9× for LINEs >5 kb), whereas TRIM28-ATRX genes were enriched for full-length LTRs (4×); most interestingly, TRIM28-FAM208A genes were enriched for LINE elements of any size but particularly those >5 kb (7×) (Fig. 3F; Supplemental Fig. S3D) and for satellites (2×). In fact, 81.72% of TRIM28-FAM208A genes contained an L1 or satellite or both within 20 kb (only TRIM28-regulated L1s were included) (Castro-Diaz et al. 2014), and L1s were often present in arrays (Fig. 3G). We verified that TRIM28-FAM208A genes contained significantly more TRIM28-L1s than random genes (e.g., 2.3× for L1Md_T) (Supplemental Fig. S3E). Because TRIM28-FAM208A genes were most enriched in LINEs >5 kb (Fig. 3F; Supplemental Fig. S3D), we selected all of these (within 20 kb), ordered them by their evolutionary age (Sookdeo et al. 2013), and found 98% to be <5 million years old and absent from rat genomes. We also ordered them by mean divergence (Supplemental Fig. S3F) and found them all to be <10% diverged from consensus sequences and the L1Md_A integrants to be the youngest by this method (mean divergence 1.53%).

In a complimentary approach, we mapped mRNA-sequencing reads to Repbase, which showed repeats to be overexpressed in all four treatment groups (Fig. 3I; Supplemental Table S5). Scoring the top five repeats derepressed in each treatment group showed that IAP elements are co-repressed by TRIM28, SETDB1, and ATRX, whereas FAM208A mainly represses young L1s (from the TF and GF families) (Supplemental Fig. S3G). In total, 25 families of repeats were co-repressed by TRIM28 and FAM208A (Fig. 3J). This included 13 L1 families that we classified by age (Sookdeo et al. 2013) and found 77% to be <3 million years old (Fig. 3K). The rest of the TRIM28-FAM208A co-repressed repeats fell into the ERV or satellite classes with satellites most highly derepressed (14×) (Fig. 3L,M). Overall, these data suggest that TRIM28 and FAM208A co-repress young L1s. Of note, we did not assess polymorphic or de novo L1 insertions.

### TRIM28-FAM208A coregulated genes are enriched in tissue-specific and new genes

**Figure 4.**
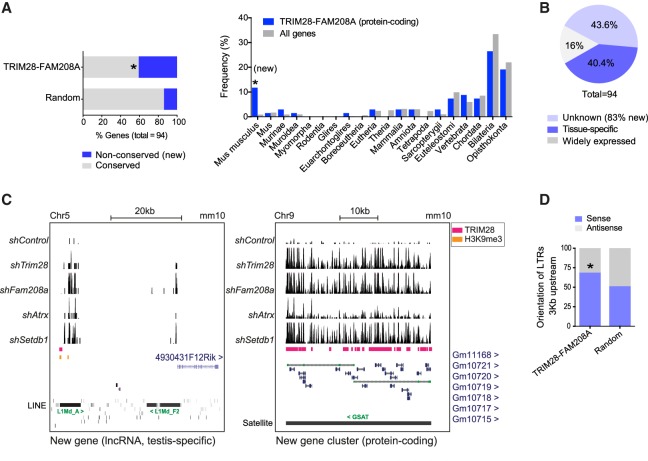
TRIM28-FAM208A coregulated genes are enriched in tissue-specific and new genes. (*A*, *left*) TRIM28-FAM208A genes and random genes were scored as conserved if they had at least 80% conservation across placental mammals (using the UCSC Table Browser). For the TRIM28-FAM208A genes, the nonconserved ones were verified to be mouse-specific using the Ensembl GeneTree. Fisher's exact test one-sided *P*-value = 2.204 × 10^−5^. (*Right*) Only protein-coding TRIM28-FAM208A genes (*n* = 68) were selected and their Last Common Ancestor extracted from the Ensembl database (version 90) using R version 3.3.1, compared to all genes in the mouse genome. Fisher's exact tests on 2 × 2 tables were significant for *Mus musculus* (*P* = 1.58 × 10^−7^). (*B*) Expression patterns of the 94 TRIM28-FAM208A genes were assessed using https://biogps.org. (*C*) mRNA-sequencing tracks of naïve J1 mESCs depleted of the stated epigenetic factors. TRIM28 peaks (Castro-Diaz et al. 2014) and TRIM28-dependent H3K9me3 (Rowe et al. 2013b) shown. See also Supplemental Figure S4. (*D*) 3 kb regions were identified upstream of each TRIM28-FAM208A coregulated gene or the random genes, and the orientation of all LTRs in these regions was assessed using the UCSC Table Browser. In the TRIM28-FAM208A group, LTRs were shown to be biased to be in a sense orientation (*P* = 0.005692, Fisher's exact one-sided test).

LTRs provide genetic material (promoters, enhancers, and first exons) to create new genes or new expression patterns (Franke et al. 2017), and active L1s can create new genes through retroposing cellular mRNAs (Carelli et al. 2016). We asked if TRIM28-FAM208A genes (Supplemental Table S6) were enriched in new genes, (which we define here as mouse-specific) and tissue-specific genes. We first found 41% of genes were not conserved across placental mammals and were mouse-specific, compared to 14% of random genes (Fig. 4A, left); focusing only on the protein-coding genes and their last common ancestor as a measure of their evolutionary age also revealed TRIM28-FAM208A genes to be enriched in mouse-specific genes (11.8% compared to 0.9% of all genes in the mouse genome) (Fig. 4A, right). Forty percent of TRIM28-FAM208A genes exhibited tissue-specific expression patterns (Fig. 4B), whereas 44% had an unknown expression pattern and this group was enriched for new genes (Fig. 4B). We verified that example loci of these new or tissue-specific genes were associated with arrays of young L1s or ERVs and epigenetic regulation (Fig. 4C; Supplemental Fig. S4). Finally, we observed that LTRs 3 kb upstream of TRIM28-FAM208A genes were biased to reside in a sense orientation (69%) suggesting they may function as promoters (Fig. 4D).

### FAM208A binds primarily to ERVs and L1 elements

**Figure 5.**
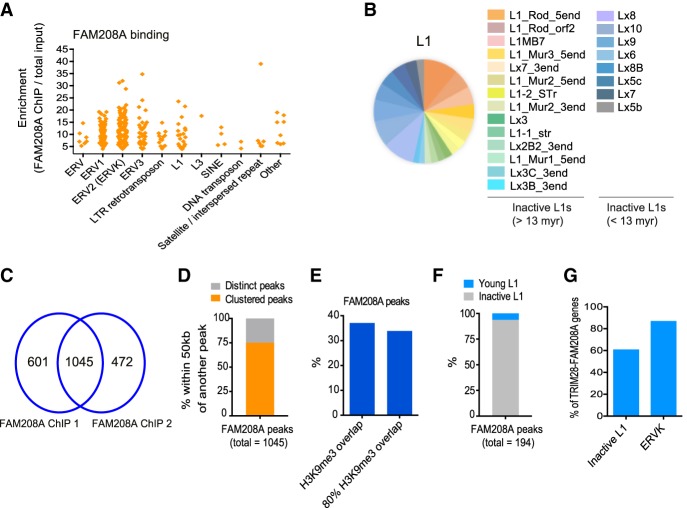
FAM208A binds primarily to ERVs and L1 elements. (*A*) Reads from TI and FAM208A IP samples were mapped to rodent Repbase. Duplicates were averaged and RPKM ratios calculated between TIs and IPs. Repeats were selected giving ≥fourfold enrichment in the IPs. The ERV2 class includes ERVK elements from ETN and IAP families. (*B*) All L1 elements from *A* are displayed here with family name and age. (*C*) After mapping reads to mm10, 1045 peaks were identified (present in both duplicates and not in the TIs). (*D*) Peaks from *C* were sorted into those that clustered by their presence within 50 kb of a second FAM208A peak. (*E*) Intersection of FAM208A peaks with H3K9me3 peaks (either any overlap or 80% overlap considered). (*F*) FAM208A peaks from *C* overlap with young versus inactive L1s. (*G*) TRIM28-FAM208A genes were assessed for the percentage that contain either an inactive L1 (from the families L1_Rod, L1MB7, L1_Mur1, L1_Mur2, L1_Mur3, Lx8, Lx9, and Lx10) or an ERVK within 20 kb.

We asked if FAM208A mainly regulates young L1s because it binds selectively to young L1s. We addressed this by performing ChIP-seq using an antibody recognizing mouse FAM208A and mapped reads to Repbase. FAM208A binds a range of retrotransposons, primarily ERVs and L1s (Fig. 5A; Supplemental Table S7), coating their 3′ halves (Supplemental Fig. S5A). Repbase L1s bound by FAM208A (22 families with an enrichment of more than 4×) were inactive families lacking full-length copies (Sookdeo et al. 2013) mostly older than 13 million years, because they were present before the mouse–rat split (Fig. 5B). We therefore also mapped reads to the genome to see if we could detect FAM208A binding to young L1s. We found 1045 FAM208A peaks (Fig. 5C; Supplemental Table S8), which clustered together (75% of ChIP-seq peaks were within 50 kb of another FAM208A peak) (Fig. 5D), suggesting FAM208A binding spreads with 34% of FAM208A peaks overlapping H3K9me3 (Fig. 5E; Rowe et al. 2013b). We could detect FAM208A binding to young L1s: Of 194 peaks targeting L1s, 6% of them were young L1s, whereas the rest were inactive L1s (Fig. 5F; for an example of a bound young L1, see Supplemental Fig. S5B). FAM208A may spread to young L1s mainly from its tethering to inactive L1s and ERVs. In support of this, we found that 61% and 87% of TRIM28-FAM208A genes (that are enriched for young L1s) (Fig. 3H) contained a FAM208A-bound L1 or an ERVK within 20 kb, respectively (Fig. 5G).

### FAM208A represses L1s in leaky heterochromatin/euchromatin

**Figure 6.**
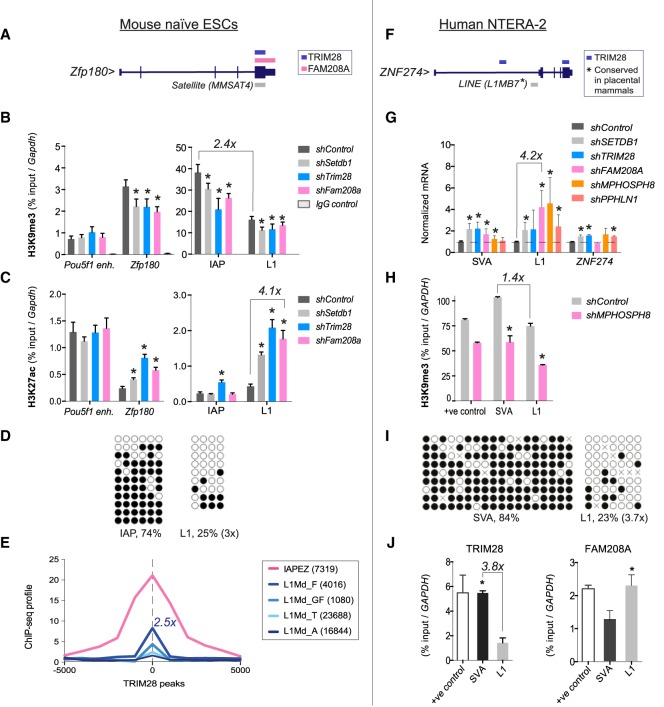
FAM208A represses L1s in leaky heterochromatin/euchromatin. (*A*) UCSC map of the *Zfp180* locus showing TRIM28 and FAM208A binding sites and overlapping repeats (see also Supplemental Fig. S6A). (*B*,*C*) H3K9me3 and H3K27ac ChIPs on naïve mESCs. Results are representative of two (in the case of H3K9me3) or three (in the case of H3K27ac) independent IPs per treatment group performed on chromatin from the same experiment (sonicated independently), and error bars show standard deviation of all IPs, each analyzed in technical triplicates by qPCR. IgG control ChIPs gave background enrichments (ranging from 0.006 to 0.043) displayed on the H3K9me3 graph. Results are normalized to *Gapdh* and the *Pou5f1* enhancer was used as an additional control region. Two-tailed unpaired *t*-tests were performed: (*) *P*-values <0.05. (*D*) DNA methylation of endogenous multicopy IAPs and L1s. (*E*) TRIM28 binding (Castro-Diaz et al. 2014) enrichment correlation with the stated repeat families using ChIP-cor. (*F*) Human *ZNF274* locus showing TRIM28 binding and the presence of a conserved L1 that is bound by FAM208A in mouse cells (Fig. 5). (*G*) qRT-PCR of retrotransposon expression (one representative experiment of three). (*H*) H3K9me3 ChIP, following *Mphosph8* depletion. Results are normalized to *GAPDH* as a negative region. +ve control; TRIM28 positive control region nearby *ZNF239* (Iyengar et al. 2011). Unpaired *t*-tests were performed: (*) *P*-values <0.05. (*I*) DNA methylation of endogenous multicopy SVAs and L1s. (*J*) ChIP-PCRs using antibodies to detect TRIM28 or FAM208A binding to SVA and L1 elements. Results are representative of two independent IPs per treatment group, and error bars show standard deviation of both IPs each analyzed in technical triplicates by qPCR. IgG control ChIPs gave only background enrichment (Supplemental Fig. S7). Results are normalized to *GAPDH*. Positive control for TRIM28: see *H*; for FAM208A, we used *TAF7*.

Mechanistic studies showed that like TRIM28 and SETDB1, FAM208A contributes to H3K9me3 maintenance at the locus *Zfp180*, which was bound by TRIM28 and FAM208A and at global IAP and L1 elements (Fig. 6A,B; Supplemental Fig. S6A). This decrease in H3K9me3 was sufficient for up-regulation of *Zfp180* (Supplemental Fig. S6B), an increase in H3K27ac (Fig. 6C), and retrotransposon reactivation (Fig. 2). The greatest shift in H3K27ac was apparent at L1s (4.1× for FAM208A) (Fig. 6C), at which we observed most derepression (13×) (Fig. 2). Of note, levels of preexisting DNA methylation were not affected in knockdown samples (Supplemental Fig. S6C). At baseline, L1s exhibited lower levels of H3K9me3 (2.4× less), DNA methylation (3× less), and TRIM28 binding (2.5× less), suggesting that they recruit “leaky” heterochromatin and as such are readily reactivated (Fig. 6B,D,E). This fits with the enrichment we observed of L1s within active TRIM28-FAM208A genes (Fig. 3F; Supplemental Fig. S3D), which recruited less H3K9me3 than TRIM28-SETDB1 genes (63% versus 77%) (Fig. 3C). Finally, we found that the HUSH complex is required to repress L1 elements and the TRIM28-repressed locus *ZNF274* in human embryonic cells, again contributing to H3K9me3 maintenance (Fig. 6F–H). L1 elements displayed lower H3K9me3 (1.4× less), DNA methylation (3.7× less), and TRIM28 binding (3.8× less) compared to SVA elements (Fig. 6H–J), perhaps explaining their adept derepression (4.2× upon *Fam208a* depletion) (Fig. 6G). Furthermore, we could detect FAM208A binding to L1 elements in human cells (Fig. 6J).

## Discussion

**Figure 7.**
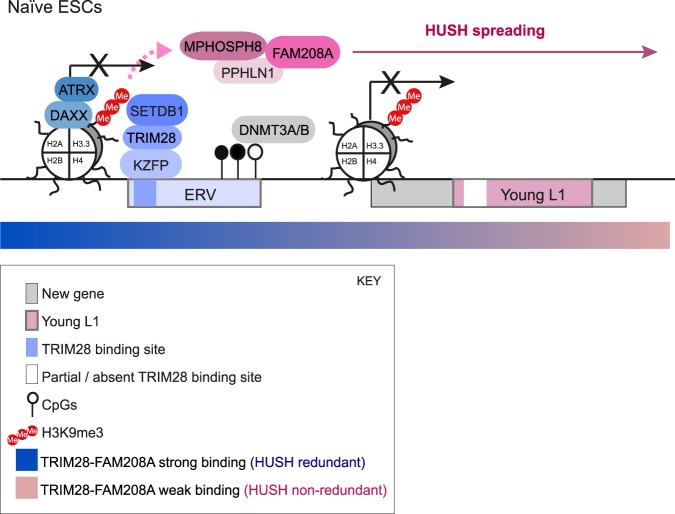
Model. ERVs recruit KZFPs, TRIM28, SETDB1, DNMT3A/B, and the H3.3/ATRX/DAXX complex. FAM208A also binds to ERVs and is known to interact with H3K9me3 through MPHOSPH8. FAM208A binding spreads through chromatin and overlaps H3K9me3, suggesting HUSH uses H3K9me3 as a platform on which to spread. TRIM28 is required to repress ERVs but FAM208A is not, likely because it is redundant at these sites of dense H3K9me3. Young L1s, in contrast, reside in “leaky heterochromatin” or part euchromatin, which exhibits weak TRIM28 and FAM208A binding and low levels of H3K9me3 and DNA methylation. Both TRIM28 and FAM208A exert nonredundant roles at young L1s. These sites are also rich in new and tissue-specific genes and are flanked by upstream sense LTRs. This suggests that genes may hijack repeats and incomplete epigenetic repression to rewire their expression patterns.

A key question has been whether the HUSH complex participates in retrotransposon repression and whether it collaborates with TRIM28 and its cofactors. Here, we show that the HUSH complex and TRIM28 exert nonredundant roles at evolutionarily young L1s of <5 million years old in naïve pluripotent cells. These young elements are likely prone to derepression because they bind TRIM28 and FAM208A only weakly. In the case of TRIM28, weak binding to young retrotransposons is known to result from them escaping KZNF recognition due to sequence divergence (Jacobs et al. 2014). In contrast, TRIM28 and FAM208A are strongly associated with ERVs, which recruit dense heterochromatin and at which only TRIM28 but not HUSH is required for repression. We found FAM208A binding spreads through chromatin, consistent with the ability of HUSH to mediate position-effect variegation (Tchasovnikarova et al. 2015). Our data reveal that TRIM28 and FAM208A coregulate new genes and tissue-specific genes, which are associated with young L1s. This suggests that regions of the genome that are enriched in young L1s and leaky heterochromatin/part euchromatin may be hot spots for the evolution of new genes and new regulation of existing genes. Such genes may hijack incomplete epigenetic repression to gain tissue-specific expression (for a summary model, see Fig. 7). TRIM28 and FAM208A cooption into these gene regulatory networks, therefore, appears to be a byproduct of their leaky regulation of young retrotransposons.

In summary, this work illustrates the complexity of epigenetic repression pathways that have evolved to regulate cellular genes through retrotransposon sequences. Future work focused on specific retrotransposon integrants will enable us to understand how these elements have been coerced to roles in gene regulation and chromatin organization in mammalian cells.

## Methods

### Cell culture

Human embryonal NTERA-2 cells (NT2/D1, a kind gift from Peter Andrews, University of Sheffield) were grown in Dulbecco's Modified Eagle's Medium (DMEM, Gibco) high glucose with 2 mM L-Glutamine, 10% fetal calf serum (FCS), and 1% Penicillin/Streptomycin (P/S). They were split in half by cell scraping. 293T cells were grown in standard DMEM + 10% FCS and P/S. J1 ESCs (*129S4/SvJae*) or their derived triple knockout (TKO) cells, which are knockout for *Dnmt3a*, *Dnmt3b*, and *Dnmt1* (from M. Okano) were used where stated, or as an independent mESC line, ES3 ESCs (Rowe et al. 2010). All mESCs were cultured on gelatin-coated plates (0.2%), either in standard media as previously described (Rowe et al. 2013a), or in media containing two small-molecule inhibitors of MEK and GSK3 plus LIF (2i + LIF), as described (Ying et al. 2008; Ficz et al. 2013). Cells were split every other day using accutase (Gibco, A11105-01) and cultured in 2i media for 7 d before transduction.

### Intracellular POU5F1 staining

Cells (1 × 10^6^ per condition) were fixed and permeabilized using intracellular staining buffers (eBioscience, 00-5523) and stained with POU5F1-PE (eBioscience, 12-5841) or isotype control (eBioscience, 12-4321) antibodies, washed, and analyzed by flow cytometry.

### Luciferase assays

Dual luciferase assays were performed at ratios detailed elsewhere (Jacobs et al. 2014). 293T cells were plated at 5 × 10^4^ cells per well in a 24-well plate and transfected with 200 ng of KZNF plasmid, 20 ng of luciferase reporter plasmid, and 2 ng of pRT-TK_*Renilla* luciferase-encoding control plasmid using 1.5 µL of FuGENE 6 (Promega) per well in triplicate wells (or for ESCs, we used 500 ng KZNF, 50 ng luciferase reporter, and 5 ng *Renilla* luciferase plasmid in 12-well plates). Forty-eight hours post-transfection, cells were lysed, and luciferase was measured using the Dual Luciferase assay kit (Promega, E1910), an opti-plate, and a GloMax 96 microplate Luminometer (Promega) using the Dual Glow program. Raw luciferase values were normalized to *Renilla* luciferase values to control transfection efficiency.

### RNA extraction and quantification

Total RNA was extracted using an RNeasy mini kit (Qiagen), treated with DNase (Ambion AM1907), and 500 ng was reverse transcribed using SuperScript II (Thermo Fisher Scientific) and random primers following the manufacturer's instructions. Samples were run on an ABI 7500 Real Time PCR System (Applied Biosystems) using SYBR green Fast PCR mastermix (Life Technologies). CT values for test genes were normalized to *B2M* and *GAPDH* for human genes or *Cox6a1* and *Gapdh* for mouse genes using the −ΔΔ*C*_t_ method to calculate fold change. See Supplemental Table S1 for primers.

### Western blotting

293T cells were washed with PBS and lysed in 2X Sample Buffer (Invitrogen) supplemented with 5% β-mercaptoethanol (Sigma). ESCs were washed 2× in ice-cold PBS and resuspended in radioimmunoprecipitation buffer with protease inhibitors added (Roche 11836170001) and quantified using the BCA Assay (Pierce). Ten micrograms was loaded per well on mini Tris/glycine gels, run in Tris/glycine/SDS buffer and mini-PROTEAN tanks (Bio-Rad) or on precast Bis-Tris gels (4%–12% or 8% gels), run in MOPs SDS buffer, followed by wet transfers to PVDF membranes. Primary antibodies are in Supplemental Table S2. Secondary antibodies were horseradish peroxidase-conjugated (GE healthcare), and membranes were developed using ECL kits (Amersham).

### Plasmids and lentiviral vectors

The luciferase reporter plasmids named Empty (pGL4cp-OCT4Enh-SV40), SVA_VNTR (pGL4cp-VNTR OCT4Enh E2), and L1PA4 (pGL4cp-L1PA4 OCT4Enh E2), and the human KZNF expression plasmids ZNF91 (pCAG ZNF91 HA) and ZNF93 (pCAG ZNF93) were a kind gift from David Haussler (Jacobs et al. 2014). Dual promoter lentiviral vectors were used for RNAi, encoding both hairpin and puromycin resistance gene (either a HIV SIREN backbone was used for human cells, from Greg Towers, or pLKO.1 for mouse cells from Dharmacon or Sigma-Aldrich). Hairpin sequences were designed (http://bioinfo.clontech.com/rnaidesigner/sirnaSequenceDesignInit.do) and annealed and cloned into *BamHI-EcoRI* sites. The shRNA pLKO.1 plasmid for SETDB1 was from Miguel Branco. See Supplemental Table S3 for shRNA sequences. VSV-G-pseudotyped lentiviral vectors were produced by FuGENE 6 (Promega) cotransfection of 293T cells in 10-cm plates with 1.5 µg shRNA-encoding plasmid, 1 µg p8.91, and 1 µg pMDG2 encoding VSV-G. The harvested supernatant was used unconcentrated for cell lines or concentrated by ultracentrifugation (20,000*g* for 2 h at 4°C) for primary cells.

### mRNA-sequencing

Mouse 2i + LIF cultured J1 ESCs treated with different shRNA vectors were used for mRNA-sequencing. Cells were cultured for 5 d following puromycin selection before RNA extraction, and samples from three independent experiments were used. See Supplemental Methods for further details.

### Repbase analysis

Reads were mapped to rodent Repbase (https://www.girinst.org/downloads/) and the latest release downloaded (Repbase 20.06 used here). The SAMtools v.1.19 idxstats utility (Li et al. 2009) was used to extract the number of mapped reads per repeat, that were inputed into the R package DESeq2 (https://bioconductor.org/packages/3.2/bioc/html/DESeq2.html) to identify differentially expressed repeats between samples depleted of epigenetic modifiers and controls, as previously described (Love et al. 2014). *P*-values were adjusted for multiple testing with the Benjamini-Hochberg false discovery rate (FDR) procedure.

### Chromatin Immunoprecipitation (ChIP)

293T cells were harvested using trypsin, whereas NTERA-2 cells and 2i + LIF grown mESCs were harvested using accutase, and chromatin was cross-linked, quenched, and prepared as described (Rowe et al. 2013b), except that sonication was performed on a Bioruptor (Diagenode). See Supplemental Methods for further details.

### DNA methylation analysis

DNA was purified with a DNeasy Blood and Tissue Kit (Qiagen), and 1 µg DNA was used for bisulfite conversion using an EpiTect Bisulfite Kit (Qiagen). Four microliters converted DNA was amplified by PCR using primers in Supplemental Table S1, which are from Rowe et al. (2013a) or were designed using http://urogene.org/methprimer/, and PCR products were cloned using the TOPO TA-Cloning Kit (Thermo Fisher Scientific), and at least 10 colonies were sent for sequencing using the T7P primer. Results were analyzed using the QUMA online tool (http://quma.cdb.riken.jp) from the Riken Institute.

### Statistical analysis

All data in the figures are presented as the standard deviation (where there are three or more samples) or by standard error of the mean (SEM) and assessed by unpaired or paired two-tailed Student *t*-tests (see figure legends for details). A *P*-value of <0.05 was considered statistically significant (***P < 0.001; **P < 0.01; *P < 0.05).

## Data access

mRNA-sequencing and ChIP-sequencing data and processed files from this study have been submitted to the NCBI Gene Expression Omnibus (GEO; http://www.ncbi.nlm.nih.gov/geo/) under accession number GSE107840, and processed files are also included in Supplemental Material.

## Supplementary Material

Supplemental Material

## References

[GR228171ROBC1] Barde I, Laurenti E, Verp S, Groner AC, Towne C, Padrun V, Aebischer P, Trumpp A, Trono D. 2009 Regulation of episomal gene expression by KRAB/KAP1-mediated histone modifications. J Virol 83: 5574–5580. 19279087 10.1128/JVI.00001-09 PMC2681943

[GR228171ROBC02] Benjamini Y, Hochberg Y. 1995 Controlling the false discovery rate: a practical and powerful approach to multiple testing. J R Statist Soc Ser B 57: 289–300.

[GR228171ROBC2] Brummelkamp TR, van Steensel B. 2015 GENE REGULATION. A HUSH for transgene expression. Science 348: 1433–1434. 26113708 10.1126/science.aac6529

[GR228171ROBC3] Cammas F, Mark M, Dolle P, Dierich A, Chambon P, Losson R. 2000 Mice lacking the transcriptional corepressor TIF1β are defective in early postimplantation development. Development 127: 2955–2963. 10851139 10.1242/dev.127.13.2955

[GR228171ROBC4] Carelli FN, Hayakawa T, Go Y, Imai H, Warnefors M, Kaessmann H. 2016 The life history of retrocopies illuminates the evolution of new mammalian genes. Genome Res 26: 301–314. 26728716 10.1101/gr.198473.115 PMC4772013

[GR228171ROBC5] Castro-Diaz N, Ecco G, Coluccio A, Kapopoulou A, Yazdanpanah B, Friedli M, Duc J, Jang SM, Turelli P, Trono D. 2014 Evolutionally dynamic L1 regulation in embryonic stem cells. Genes Dev 28: 1397–1409. 24939876 10.1101/gad.241661.114 PMC4083085

[GR228171ROBC6] Diederichs S, Bartsch L, Berkmann JC, Frose K, Heitmann J, Hoppe C, Iggena D, Jazmati D, Karschnia P, Linsenmeier M, 2016 The dark matter of the cancer genome: aberrations in regulatory elements, untranslated regions, splice sites, non-coding RNA and synonymous mutations. EMBO Mol Med 8: 442–457. 26992833 10.15252/emmm.201506055 PMC5126213

[GR228171ROBC7] Dodge JE, Kang YK, Beppu H, Lei H, Li E. 2004 Histone H3-K9 methyltransferase ESET is essential for early development. Mol Cell Biol 24: 2478–2486. 14993285 10.1128/MCB.24.6.2478-2486.2004 PMC355869

[GR228171ROBC8] Elsasser SJ, Noh KM, Diaz N, Allis CD, Banaszynski LA. 2015 Histone H3.3 is required for endogenous retroviral element silencing in embryonic stem cells. Nature 522: 240–244. 25938714 10.1038/nature14345 PMC4509593

[GR228171ROBC9] Ficz G, Hore TA, Santos F, Lee HJ, Dean W, Arand J, Krueger F, Oxley D, Paul YL, Walter J, 2013 FGF signaling inhibition in ESCs drives rapid genome-wide demethylation to the epigenetic ground state of pluripotency. Cell Stem Cell 13: 351–359. 23850245 10.1016/j.stem.2013.06.004 PMC3765959

[GR228171ROBC10] Franke V, Ganesh S, Karlic R, Malik R, Pasulka J, Horvat F, Kuzman M, Fulka H, Cernohorska M, Urbanova J, 2017 Long terminal repeats power evolution of genes and gene expression programs in mammalian oocytes and zygotes. Genome Res 27: 1384–1394. 28522611 10.1101/gr.216150.116 PMC5538554

[GR228171ROBC11] Garrick D, Sharpe JA, Arkell R, Dobbie L, Smith AJ, Wood WG, Higgs DR, Gibbons RJ. 2006 Loss of Atrx affects trophoblast development and the pattern of X-inactivation in extraembryonic tissues. PLoS Genet 2: e58. 16628246 10.1371/journal.pgen.0020058 PMC1440874

[GR228171ROBC12] Harten SK, Bruxner TJ, Bharti V, Blewitt M, Nguyen TM, Whitelaw E, Epp T. 2014 The first mouse mutants of *D14Abb1e* (*Fam208a*) show that it is critical for early development. Mamm Genome 25: 293–303. 24781204 10.1007/s00335-014-9516-0 PMC4105592

[GR228171ROBC13] He Q, Kim H, Huang R, Lu W, Tang M, Shi F, Yang D, Zhang X, Huang J, Liu D, 2015 The Daxx/Atrx complex protects tandem repetitive elements during DNA hypomethylation by promoting H3K9 trimethylation. Cell Stem Cell 17: 273–286. 26340527 10.1016/j.stem.2015.07.022 PMC4571182

[GR228171ROBC14] Hummel B, Hansen EC, Yoveva A, Aprile-Garcia F, Hussong R, Sawarkar R. 2017 The evolutionary capacitor HSP90 buffers the regulatory effects of mammalian endogenous retroviruses. Nat Struct Mol Biol 24: 234–242. 28134929 10.1038/nsmb.3368

[GR228171ROBC15] Imbeault M, Helleboid PY, Trono D. 2017 KRAB zinc-finger proteins contribute to the evolution of gene regulatory networks. Nature 543: 550–554. 28273063 10.1038/nature21683

[GR228171ROBC16] Ivanov AV, Peng H, Yurchenko V, Yap KL, Negorev DG, Schultz DC, Psulkowski E, Fredericks WJ, White DE, Maul GG, 2007 PHD domain-mediated E3 ligase activity directs intramolecular sumoylation of an adjacent bromodomain required for gene silencing. Mol Cell 28: 823–837. 18082607 10.1016/j.molcel.2007.11.012 PMC4348069

[GR228171ROBC17] Iyengar S, Ivanov AV, Jin VX, Rauscher FJ III, Farnham PJ. 2011 Functional analysis of KAP1 genomic recruitment. Mol Cell Biol 31: 1833–1847. 21343339 10.1128/MCB.01331-10 PMC3133220

[GR228171ROBC18] Jacobs FM, Greenberg D, Nguyen N, Haeussler M, Ewing AD, Katzman S, Paten B, Salama SR, Haussler D. 2014 An evolutionary arms race between KRAB zinc-finger genes *ZNF91/93* and SVA/L1 retrotransposons. Nature 516: 242–245. 25274305 10.1038/nature13760 PMC4268317

[GR228171ROBC19] Karimi MM, Goyal P, Maksakova IA, Bilenky M, Leung D, Tang JX, Shinkai Y, Mager DL, Jones S, Hirst M, 2011 DNA methylation and SETDB1/H3K9me3 regulate predominantly distinct sets of genes, retroelements, and chimeric transcripts in mESCs. Cell Stem Cell 8: 676–687. 21624812 10.1016/j.stem.2011.04.004 PMC3857791

[GR228171ROBC20] Kokura K, Sun L, Bedford MT, Fang J. 2010 Methyl-H3K9-binding protein MPP8 mediates E-cadherin gene silencing and promotes tumour cell motility and invasion. EMBO J 29: 3673–3687. 20871592 10.1038/emboj.2010.239 PMC2982762

[GR228171ROBC21] Lechner MS, Begg GE, Speicher DW, Rauscher FJ III. 2000 Molecular determinants for targeting heterochromatin protein 1-mediated gene silencing: Direct chromoshadow domain–KAP-1 corepressor interaction is essential. Mol Cell Biol 20: 6449–6465. 10938122 10.1128/mcb.20.17.6449-6465.2000 PMC86120

[GR228171ROBC22] Lemaitre C, Bickmore WA. 2015 Chromatin at the nuclear periphery and the regulation of genome functions. Histochem Cell Biol 144: 111–122. 26170147 10.1007/s00418-015-1346-y

[GR228171ROBC23] Li H, Handsaker B, Wysoker A, Fennell T, Ruan J, Homer N, Marth G, Abecasis G, Durbin R; Genome Project Data Processing Subgroup. 2009 The Sequence Alignment/Map format and SAMtools. Bioinformatics 25: 2078–2079. 19505943 10.1093/bioinformatics/btp352 PMC2723002

[GR228171ROBC24] Lippman Z, Gendrel AV, Black M, Vaughn MW, Dedhia N, McCombie WR, Lavine K, Mittal V, May B, Kasschau KD, 2004 Role of transposable elements in heterochromatin and epigenetic control. Nature 430: 471–476. 15269773 10.1038/nature02651

[GR228171ROBC25] Love MI, Huber W, Anders S. 2014 Moderated estimation of fold change and dispersion for RNA-seq data with DESeq2. Genome Biol 15: 550. 25516281 10.1186/s13059-014-0550-8 PMC4302049

[GR228171ROBC26] Matsui T, Leung D, Miyashita H, Maksakova IA, Miyachi H, Kimura H, Tachibana M, Lorincz MC, Shinkai Y. 2010 Proviral silencing in embryonic stem cells requires the histone methyltransferase ESET. Nature 464: 927–931. 20164836 10.1038/nature08858

[GR228171ROBC27] Rebollo R, Karimi MM, Bilenky M, Gagnier L, Miceli-Royer K, Zhang Y, Goyal P, Keane TM, Jones S, Hirst M, 2011 Retrotransposon-induced heterochromatin spreading in the mouse revealed by insertional polymorphisms. PLoS Genet 7: e1002301. 21980304 10.1371/journal.pgen.1002301 PMC3183085

[GR228171ROBC28] Robbez-Masson L, Rowe HM. 2015 Retrotransposons shape species-specific embryonic stem cell gene expression. Retrovirology 12: 45. 26021318 10.1186/s12977-015-0173-5 PMC4448215

[GR228171ROBC29] Rowe HM, Jakobsson J, Mesnard D, Rougemont J, Reynard S, Aktas T, Maillard PV, Layard-Liesching H, Verp S, Marquis J, 2010 KAP1 controls endogenous retroviruses in embryonic stem cells. Nature 463: 237–240. 20075919 10.1038/nature08674

[GR228171ROBC30] Rowe HM, Friedli M, Offner S, Verp S, Mesnard D, Marquis J, Aktas T, Trono D. 2013a *De novo* DNA methylation of endogenous retroviruses is shaped by KRAB-ZFPs/KAP1 and ESET. Development 140: 519–529. 23293284 10.1242/dev.087585 PMC4892343

[GR228171ROBC31] Rowe HM, Kapopoulou A, Corsinotti A, Fasching L, Macfarlan TS, Tarabay Y, Viville S, Jakobsson J, Pfaff SL, Trono D. 2013b TRIM28 repression of retrotransposon-based enhancers is necessary to preserve transcriptional dynamics in embryonic stem cells. Genome Res 23: 452–461. 23233547 10.1101/gr.147678.112 PMC3589534

[GR228171ROBC32] Sadic D, Schmidt K, Groh S, Kondofersky I, Ellwart J, Fuchs C, Theis FJ, Schotta G. 2015 Atrx promotes heterochromatin formation at retrotransposons. EMBO Rep 16: 836–850. 26012739 10.15252/embr.201439937 PMC4515123

[GR228171ROBC33] Sanjana NE, Wright J, Zheng K, Shalem O, Fontanillas P, Joung J, Cheng C, Regev A, Zhang F. 2016 High-resolution interrogation of functional elements in the noncoding genome. Science 353: 1545–1549. 27708104 10.1126/science.aaf7613 PMC5144102

[GR228171ROBC34] Schmitges FW, Radovani E, Najafabadi HS, Barazandeh M, Campitelli LF, Yin Y, Jolma A, Zhong G, Guo H, Kanagalingam T, 2016 Multiparameter functional diversity of human C2H2 zinc finger proteins. Genome Res 26: 1742–1752. 27852650 10.1101/gr.209643.116 PMC5131825

[GR228171ROBC35] Sookdeo A, Hepp CM, McClure MA, Boissinot S. 2013 Revisiting the evolution of mouse LINE-1 in the genomic era. Mob DNA 4: 3. 23286374 10.1186/1759-8753-4-3 PMC3600994

[GR228171ROBC36] Tchasovnikarova IA, Timms RT, Matheson NJ, Wals K, Antrobus R, Göttgens B, Dougan G, Dawson MA, Lehner PJ. 2015 GENE SILENCING. Epigenetic silencing by the HUSH complex mediates position-effect variegation in human cells. Science 348: 1481–1485. 26022416 10.1126/science.aaa7227 PMC4487827

[GR228171ROBC37] Tchasovnikarova IA, Timms RT, Douse CH, Roberts RC, Dougan G, Kingston RE, Modis Y, Lehner PJ. 2017 Hyperactivation of HUSH complex function by Charcot–Marie–Tooth disease mutation in *MORC2*. Nat Genet 49: 1035–1044. 28581500 10.1038/ng.3878 PMC5493197

[GR228171ROBC38] Thompson PJ, Macfarlan TS, Lorincz MC. 2016 Long terminal repeats: from parasitic elements to building blocks of the transcriptional regulatory repertoire. Mol Cell 62: 766–776. 27259207 10.1016/j.molcel.2016.03.029 PMC4910160

[GR228171ROBC39] Timms RT, Tchasovnikarova IA, Antrobus R, Dougan G, Lehner PJ. 2016 ATF7IP-mediated stabilization of the histone methyltransferase SETDB1 is essential for heterochromatin formation by the HUSH complex. Cell Rep 17: 653–659. 27732843 10.1016/j.celrep.2016.09.050 PMC5081395

[GR228171ROBC40] Turelli P, Castro-Diaz N, Marzetta F, Kapopoulou A, Raclot C, Duc J, Tieng V, Quenneville S, Trono D. 2014 Interplay of TRIM28 and DNA methylation in controlling human endogenous retroelements. Genome Res 24: 1260–1270. 24879559 10.1101/gr.172833.114 PMC4120080

[GR228171ROBC41] Wolf D, Goff SP. 2009 Embryonic stem cells use ZFP809 to silence retroviral DNAs. Nature 458: 1201–1204. 19270682 10.1038/nature07844 PMC2676211

[GR228171ROBC42] Wolf G, Rebollo R, Karimi MM, Ewing AD, Kamada R, Wu W, Wu B, Bachu M, Ozato K, Faulkner GJ, 2017 On the role of H3.3 in retroviral silencing. Nature 548: E1–E3. 28770848 10.1038/nature23277 PMC6258051

[GR228171ROBC43] Yang BX, El Farran CA, Guo HC, Yu T, Fang HT, Wang HF, Schlesinger S, Seah YF, Goh GY, Neo SP, 2015 Systematic identification of factors for provirus silencing in embryonic stem cells. Cell 163: 230–245. 26365490 10.1016/j.cell.2015.08.037 PMC4686136

[GR228171ROBC44] Ying QL, Wray J, Nichols J, Batlle-Morera L, Doble B, Woodgett J, Cohen P, Smith A. 2008 The ground state of embryonic stem cell self-renewal. Nature 453: 519–523. 18497825 10.1038/nature06968 PMC5328678

