# Supplementary material for: The HUSH complex cooperates with TRIM28 to repress young retrotransposons and new genes
Source: Genome Res. 2018 Jun;28(6):836–45. doi: 10.1101/gr.228171.117 (PMC5991525; doi:10.1101/gr.228171.117)
Supplement: Supplemental Material [file supp_28_6_836__index.html]

The HUSH complex cooperates with TRIM28 to repress young retrotransposons and new genes — Supplemental Material 

# The HUSH complex cooperates with TRIM28 to repress young retrotransposons and new genes

## Supplemental Material

- Supplemental\_Methods.pdf
- Supplemental\_Table\_S1.docx
- Supplemental\_Table\_S2.docx
- Supplemental\_Table\_S3.docx
- Supplemental\_Table\_S4.xls
- Supplemental\_Table\_S5.xls
- Supplemental\_Table\_S6.xlsx
- Supplemental\_Table\_S7.xls
- Supplemental\_Table\_S8.xls
- Supplemental\_Figures\_Legends.pdf
