# Supplementary material for: The HUSH complex cooperates with TRIM28 to repress young retrotransposons and new genes
Source: Genome Res. 2018 Jun;28(6):836–45. doi: 10.1101/gr.228171.117 (PMC5991525; doi:10.1101/gr.228171.117)
Supplement: Supplemental Material [file supp_gr.228171.117_Supplemental_Methods.pdf]

Supplemental Methods

mRNA-sequencing and analysis

The RNA was quality checked on a 2200 Tapestation using the RNA ScreenTape assay (Agilent Technologies, Wokingham, UK) and the RNA concentration measured using a Qubit RNA High Sensitivity kit (LifeTechnologies, Paisley, UK). PolyA-tailed mRNA was separated for sequencing during library preparation. Libraries were prepared using KAPA's Stranded mRNASeq kit (Roche Diagnostics, Burgess Hill, UK) according to the manufacturer's instructions using an input of 500 ng, a fragmentation incubation time of 6 minutes at 94°C, and 12 cycles of PCR for library amplification. Samples were sequenced on Illumina's NextSeq500 (Illumina Cambridge, Chesterford, UK) using a high output 300 cycle paired-end run. Twenty-four libraries were multiplexed in the same run to achieve up to 16M reads/sample. Libraries were pooled in equimolar quantities, calculated from concentrations measured using the Qubit dsDNA High Sensitivity assay kit (Life Technologies, Paisley, UK) and fragment analysis using the D1000 High Sensitivity kit on the 2200 Tapestation (Agilent Technologies, Wokingham, UK). For the analysis, reads were mapped against the mouse transcriptome (GRCm38/mm10, rel 79) using Kallisto (https://pachterlab.github.io/kallisto/) with default parameters (k-mers equal to 31). Data were imported into R using the R package (tximport) and differentially expressed genes were identified with DESeq2 (https://f1000research.com/articles/4-1521/v1). P-values were adjusted for multiple testing with the Benjamini-Hochberg false-discovery-rate (FDR). In order to generate bigWig files to view on the genome browser, reads were first mapped to the genome using Bowtie 2 v2.2.4 (Langmead and Salzberg 2012), which reports the best read alignment and assigns the read randomly to one location if two matches are equally as good. This is one approach for

mapping repetitive regions. BAM files were then converted to bigWig files, which were generated as URL links. Reads were mapped to rodent RepBase (http://www.girinst.org/server/RepBase/protected/RepBase20.06.fasta/rodrep.ref). The samtools v.1.19 idxstats utility was used to extract the number of mapped reads per repeat, that were inputed into the R package DESeq2 (https://bioconductor.org/packages/3.2/bioc/html/DESeq2.html) to identify differentially expressed repeats between samples depleted of epigenetic modifiers and controls (as previously described (Love et al. 2014)). P-values were adjusted for multiple testing with the Benjamini-Hochberg false-discovery-rate (FDR) procedure.

Chromatin immunoprecipitation (ChIP) and analysis

Samples were sonicated at $10x10^6$ cells per tube in 300ul lysis buffer for 15 to 20 cycles of 30sec ON/30sec OFF, high power, and fragment size was verified on 2% agarose gels. Chromatin was precleared with beads (protein G dynabeads, 10003D) before immunoprecipitations (IPs) with rabbit antibodies against H3K9me3 (Abcam, ab8898) H3K27ac (Abcam, ab4729), TRIM28 (Abcam, ab10483) and FAM208A (TASOR) (Atlas, HPA006735 for human cells and Atlas, HPA017142 for mouse cells), which were all performed with $3x10^6$ cells per IP. 5ug of antibody was used per IP except for TRIM28 and FAM208A where 10ug was used. After reverse-crosslinking, DNA was purified with a minElute PCR purification kit (Qiagen). Quantification by qPCR was performed on an ABI 7500 Real Time PCR System (Applied Biosystems) using SYBR green FAST PCR mastermix (Life Technologies). Ct values of IPs were normalized to their total input (TI) samples. See Supplemental Table S1 for primer sequences.

ChIP-sequencing was performed for FAM208A samples (duplicate IPs and duplicate TIs) by UCL Genomics using a fragment size selection of 150 – 300bp for library preparation (NEBNext DNA Ultra II library prep kit). 1ng was used and 10 cycles of amplification. Sequencing was on a NextSeq500 (single 86bp read sequencing with >100 million reads per sample). Trim_galore v0.4.1 was used to remove adapters and for trimming reads. Reads were mapped against mm10 using Bowtie 2-2.2.4 with default parameters and duplicates were removed with picard-1.135. FAM208A peaks were called using MACS2 v2.1.1.20160309 with --nomodel parameter, extension size = 150 and total Inputs as controls. Consensus peaks between FAM208A replicates were derived using Diffbind_2.4.8, and overlap of peaks with genes and repeats were obtained using bedtools-2.17.0 on mm10 data downloaded from the UCSC browser. Chip-seq reads were also mapped to RepBase20.06/rodent.ref (see above for RNA-sequencing). The SAMtools v.1.19 idxstats and flgstat utilities (Li et al. 2009) were used to extract the number of mapped reads per repeat and calculate RPKMs in each sample.

References

Langmead B, Salzberg SL. 2012. Fast gapped-read alignment with Bowtie 2. *Nat Methods* **9**: 357-359.

Li H, Handsaker B, Wysoker A, Fennell T, Ruan J, Homer N, Marth G, Abecasis G, Durbin R, Genome Project Data Processing S. 2009. The Sequence Alignment/Map format and SAMtools. *Bioinformatics* **25**: 2078-2079.

Love MI, Huber W, Anders S. 2014. Moderated estimation of fold change and dispersion for RNA-seq data with DESeq2. *Genome Biol* **15**: 550.
