# Supplementary material for: The HUSH complex cooperates with TRIM28 to repress young retrotransposons and new genes
Source: Genome Res. 2018 Jun;28(6):836–45. doi: 10.1101/gr.228171.117 (PMC5991525; doi:10.1101/gr.228171.117)
Supplement: Supplemental Material [file supp_gr.228171.117_Supplemental_Table_S1.docx]

Supplemental Table S1: Primers

**qRT-PCR**

| *Primer name* | *Forward* | *Reverse* | *species* |
| --- | --- | --- | --- |
| ***TRIM28 (KAP1)*** | **AAGGACCATACTGTGCGCTCTAC** | **ACGTTGCAATAGACAGTACGTTCAC** | **Human** |
| ***SETDB1*** | **CAAGCTGGGACTACAATACCG** | **TCTGGTCTTTTGGAGTTCTGC** | **Human** |
| ***ATRX*** | **CCATTTATAGACACCCTTCATTGC** | **CAAATCAAGTTTCCACCTTCCG** | **Human** |
| ***DAXX*** | **AGAAGCAAACAGGATCAGGG** | **GCTGGGCAGGGTACATATC** | **Human** |
| ***H3F3A (H3.3)*** | **GGAAGCAACTGGCTACAAAAG** | **GGGAAGTTTGCGAATCAGAAG** | **Human** |
| ***MPHOSPH8 (MPP8)*** | **TGCCTGTATCTGCCCAAAC** | **CCTTGTCAGAATCATGCCTTTTC** | **Human** |
| ***PPHLN1 (Periphilin)*** | **GGTTCCAGTGTCAGTAGCAG** | **TCATTCTGCCGTTTGTAGGAG** | **Human** |
| ***FAM208A (TASOR)*** | **TGAAGACATTGCAGGTTTCATTC** | **CATCCAGGCTATCAACACCAG** | **Human** |
| ***CBX5 (HP1 alpha)*** | **CCAATTTCTCAAACAGTGCCG** | **GTTGCCCCAATGATCTTTTCTG** | **Human** |
| ***CBX1 (HP1 beta)*** | **TGGTAAAGGGCAAAGTGGAG** | **CATGTGCTGTTTTCTGTGACTG** | **Human** |
| ***CBX3 (HP1 gamma)*** | **GGCCTCCAACAAAACTACATTG** | **TCCACTTTCCCATTCACTACAC** | **Human** |
| ***ZNF274*** | **ACACAGAGGACCGAGTACC** | **TTCTTGTACTTTCAGGCTGGG** | **Human** |
| ***ZNF91*** | **GACTTTTGGCCAGAGCAGAG** | **CTCTGGGCAGTTGTGAGACA** | **Human** |
| ***ZNF93*** | **AGAAGCCCTACGTTTGTGAAG** | **GGTTGAGGATGCAATAAAGGC** | **Human** |
| ***B2M*** | **TGCTCGCGCTACTCTCTCTTT** | **TCTGCTGGATGACGTGAGTAAAC** | **Human** |
| ***GAPDH*** | **ACATCGCTCAGACACCATG** | **TGTAGTTGAGGTCAATGAAGGG** | **Human** |
| ***SVA (541 loci)*** | **CTCGTTCACTCAGTGCTCAATG** | **CTGGGAGGTGGAGGTTGTAG** | **Human** |
| ***L1 (2102 loci)*** | **GAACGCCACAAAGATACTCC** | **CTCTTCTGGCTTGTAGGGTTTCTG** | **Human** |
| ***Zfp180*** | **CCAGAGAACCCCAGAGAAAAC** | **CCCCTCTAGCTTCAATCCATG** | **Mouse** |
| ***Prnp*** | **CCTTCCTAGTGGTACCAGTCCAA** | **AGCCAAGGTTCGCCATGA** | **Mouse** |
| ***Trim28*** | **CGGAAATGTGAGCGTGTTCTC** | **CGGTAGCCAGCTGATGCAA** | **Mouse** |
| ***Setdb1*** | **TGGCAACAGCGGTTCAGA** | **CAGAAGTTATCATCAGAGCTGTCATCA** | **Mouse** |
| ***Atrx*** | **ACACCCTTCACTCAAAGTCC** | **CAAATCAAGTTCCCACCTTCTG** | **Mouse** |
| ***H3f3a*** | **TCGGTGTCAGCCATCTTTC** | **TTTGTACGAGCCATGGTAAGG** | **Mouse** |
| ***H3f3b*** | **CGTTACCAGAAATCGACTGAGC** | **CACCAGGTATGCTTCGCTAG** | **Mouse** |
| ***Mphosph8*** | **AGCTACCATCACCTGTGTTTG** | **CCTTTTCTGGTATTTTCCTTTGGG** | **Mouse** |
| ***Fam208a*** | **CCACGGTTTCTATTGAGCATG** | **TTTGTTTCTCACCATGTTTCCC** | **Mouse** |
| ***Dnmt1*** | **CCAGGCATTTCGGCTGAA** | **CGTTGCAGTCCTCTGTGAACA** | **Mouse** |
| ***Dnmt3b*** | **AACTCCATCAGACAGGGCAAA** | **CGTCCTTGCCATTCATGACTAC** | **Mouse** |
| ***Cox6a1*** | **CTCTTCCACAACCCTCATGTGA** | **GAGGCCAGGTTCTCTTTACTCATC** | **Mouse** |
| ***Gapdh*** | **TCCATGACAACTTTGGCATTG** | **CAGTCTTCTGGGTGGCAGTGA** | **Mouse** |
| ***IAP (1086 loci)*** | **CGGGTCGCGGTAATAAAGGT** | **ACTCTCGTTCCCCAGCTGAA** | **Mouse** |
| ***ERVK (186 loci)*** | **CTTCCGTGGGTGTTAATCCTC** | **GCATGTAGGACACCAGAACTG** | **Mouse** |
| ***L1 (1330 loci)*** | **TTTGGGACACAATGAAAGCA** | **CTGCCGTCTACTCCTCTTGG** | **Mouse** |

**ChIP primers**

| *Primer name* | *Forward* | *Reverse* | *Species* |
| --- | --- | --- | --- |
| *Reporter* | AGGCTACAAACGCTCTCATC | GGTAGCCCTTGTATTTGATCAGG |  |
| *ZNF180* | TGATGCACAATAAGTCGAGCA | TGCAGTCAATGTGGGAAGTC | Human |
| *EVX1* | CTGGGTGTCTCCCTCTCTCA | AAAGGAAACCCGCAGCTAAT | Human |
| *GAPDH* | CACCGTCAAGGCTGAGAACG | ATACCCAAGGGAGCCACACC | Human |
| TRIM28 +ve CTR (nearby *ZNF239*) | GGAGAAATCCCATGAGGGTAA | GGCTTTTGTGAGAATGTTTTCC | Human |
| SVA | CTCGTTCACTCAGTGCTCAATG | CTGGGAGGTGGAGGTTGTAG | Human |
| L1 | GAACGCCACAAAGATACTCC | CTCTTCTGGCTTGTAGGGTTTCTG | Human |
| *Gapdh* | GGCCGCCGCCATGT | AGCTAGGAAGAAGGAAGGCCTAAG | Mouse |
| *Pou5f1 enhancer* | GGAGGCAAGAAACTGGATCAGA | CCTGGTGGCCCTGGAGAT | Mouse |
| *Zfp180* | CCGTACAGGTGCAATCTGTG | GTTTGTAGCTCTGGCGGAAC | Mouse |
| IAP | CGGGTCGCGGTAATAAAGGT | ACTCTCGTTCCCCAGCTGAA | Mouse |
| L1 | TTTGGGACACAATGAAAGCA | CTGCCGTCTACTCCTCTTGG | Mouse |

**DNA methylation**

| *Primer name* | *Forward* | *Reverse* | *CpG* |  |
| --- | --- | --- | --- | --- |
| SV40_4 | TGAAGGGTAGGTGGTTTTATTTTAGT | CCATAATAACTTTACCAACAATACC | 10 |  |
| L1PA4_chr12 | GGGTGATTTTTGTATTTTTAATTGAG | AAAAAAAATTCCCTAACCCCTTAC | 7 | H |
| SVA_chr7 | TTGTAATTTTTTTGTTTGATTTTTTTGT | TACACTCCAACCTAAACACCATTAA | 18 | H |
| IAP LTR | GGTTTTGGAATGAGGGATTTT | CTCTACTCCATATACTCTACCTTC | 6 | M |
| IAP 5’UTR | GGGTTGTAGTTAATTAGGGAGTGATA | ACAATTAAATCCTTCTTAACAATCTACTT | 9 | M |
| IAP575_5'LTR | TAGGTAGGGTTATTATGTGAGGTTTTA | CCCCTCCCTCTTAAAAAACATTCTCCTT | 10 | M |
| L1 | GGTTTTTAATGGAGGAGTTAGAGAAAGTA | ACTCCCCCCCTTTAACCCT | 4 | M |
| L1Md_F2_chr7 | GATGGAAATGAATAAAATTATATTAGTTTT | ATTTACTAACCCTTTTAATTAAAAATCTTC | 3 | M |
| MERVK10C | TGTTGAGAAGGATTTAATTGTATGG | AAACAAAAAAACAAACAAACAACAC | 4 | M |
