# Supplementary material for: The HUSH complex cooperates with TRIM28 to repress young retrotransposons and new genes
Source: Genome Res. 2018 Jun;28(6):836–45. doi: 10.1101/gr.228171.117 (PMC5991525; doi:10.1101/gr.228171.117)
Supplement: Supplemental Material [file supp_gr.228171.117_Supplemental_Table_S2.docx]

Supplemental Table S2: Antibodies

| β-ACTIN | Millipore, MAB1501 | mouse monoclonal |
| --- | --- | --- |
| ATRX | Santa Cruz, H-300 sc-15408 | rabbit polyclonal |
| CBX5 (HP1 alpha) | Cell Signaling, 2616 | rabbit polyclonal |
| CBX1 (HP1 beta) | Cell Signaling, 2613 | rabbit polyclonal |
| CBX3 (HP1 gamma) | Abcam, ab10480 | rabbit polyclonal |
| TRIM28 | Millipore, MAB3662 | mouse monoclonal |
| MPHOSPH8 (MPP8) | Proteintech, 16796-1-AP | rabbit polyclonal |
| NANOG | Abcam ab80892 | rabbit Polyclonal |
| PCNA | Millipore, clone PC10 | mouse monoclonal |
| SETDB1 | Proteintech 11231-1-AP | rabbit polyclonal |
| FAM208A / TASOR (human) | Atlas, HPA006735 | rabbit polyclonal |
| FAM208A / TASOR (human/mouse) | Atlas, HPA017142 | rabbit polyclonal |
