# Supplementary material for: The HUSH complex cooperates with TRIM28 to repress young retrotransposons and new genes
Source: Genome Res. 2018 Jun;28(6):836–45. doi: 10.1101/gr.228171.117 (PMC5991525; doi:10.1101/gr.228171.117)
Supplement: Supplemental Material [file supp_gr.228171.117_Supplemental_Table_S3.docx]

Supplemental Table S3: shRNAs

| Human | Hairpin sequences |
| --- | --- |
| *TRIM28* | 5'-gatccGCCTGGCTCTGTTCTCTGTCCTTTCAAGAGAAGGACAGAGAACAGAGCCAGGTTTTTTACGCGTg-----3' |
|  | 5'-aattcACGCGTAAAAAACCTGGCTCTGTTCTCTGTCCTTCTCTTGAAAGGACAGAGAACAGAGCCAGGCg-----3' |
| *FAM208A* (TASOR) | 5'_gatccGAGGAAGCTTGAGGATCTATTCAAGAGATAGATCCTCAAGCTTCCTCTTTTTTg_3’ |
|  | 5'_aattcAAAAAAGAGGAAGCTTGAGGATCTATCTCTTGAATAGATCCTCAAGCTTCCTCg_3 |
| *MPHOSPH8* (MPP8) | 5'_gatccAAGAAGACCCCGAGAAAGGTTCAAGAGACCTTTCTCGGGGTCTTCTTTTTTTTg_3’ |
|  | 5'_aattcAAAAAAAAGAAGACCCCGAGAAAGGTCTCTTGAACCTTTCTCGGGGTCTTCTTGGATCg_3’ |
| *PPHLN1* (PERIPHILIN) | 5’_gatccAGCTAACCACTCGCTCTAATTCAAGAGATTAGAGCGAGTGGTTAGCTTTTTTTg_3’ |
|  | 5’_aattcAAAAAAAGCTAACCACTCGCTCTAATCTCTTGAATTAGAGCGAGTGGTTAGCTGGATCg_3’ |
| *SETDB1* | 5'-gatccGAGGAACTGGGTATCTCTATTCAAGAGATAGAGATACCCAGTTCCTCTTTTTTACGCGTg-----3' |
|  | 5'-aattcACGCGTAAAAAAGAGGAACTGGGTATCTCTATCTCTTGAATAGAGATACCCAGTTCCTCg-----3' |
| *ATRX* | 5'-gatccTACGCAACCTTGGTCGAAATTCAAGAGATTTCGACCAAGGTTGCGTATTTTTTACGCGTg-----3' |
|  | 5'-aattcACGCGTAAAAAATACGCAACCTTGGTCGAAATCTCTTGAATTTCGACCAAGGTTGCGTACg-----3' |
| *DAXX* | 5'-gatccATGCGAGGTTCTGAGAATTTTCAAGAGAAATTCTCAGAACCTCGCATTTTTTTACGCGTg-----3' |
|  | 5'-aattcACGCGTAAAAAAATGCGAGGTTCTGAGAATTTCTCTTGAAAATTCTCAGAACCTCGCATg-----3' |
| *H3F3A (H3.3)* | 5'-gatccGCGAGAAATTGCTCAGGACTTTCAAGAGAAGTCCTGAGCAATTTCTCGTTTTTTACGCGTg-----3' |
|  | 5'-aattcACGCGTAAAAAACGAGAAATTGCTCAGGACTTCTCTTGAAAGTCCTGAGCAATTTCTCGCg-----3' |
|  |  |
| Mouse * |  |
| *Trim28* | TRCN0000071363 |
| *Setdb1* | TRCN0000092975 |
| *Atrx* | TRCN0000081909 |
| *H3f3a* | TRCN0000012027 |
| *H3f3b* | TRCN0000092918 |
| *H3f3b* | TRCN0000092919 |
| *H3f3b* | TRCN0000092920 |
| *Fam208a* (TASOR) | TRCN0000251275 |
| *Mphosph8* (MPP8) | TRCN0000085438 |
| *Mphosph8* (MPP8) | TRCN0000085439 |
|  |  |
| * shRNA vectors were commercial PLKO.1 vectors. | |
| Hairpins for *H3f3b* were tested as a pool and the best hairpin not determined. | |
| For MPHOSPH8, both hairpins worked well. | |
