# Supplementary material for: The HUSH complex cooperates with TRIM28 to repress young retrotransposons and new genes
Source: Genome Res. 2018 Jun;28(6):836–45. doi: 10.1101/gr.228171.117 (PMC5991525; doi:10.1101/gr.228171.117)
Supplement: Supplemental Material [file supp_gr.228171.117_Supplemental_Figures_Legends.pdf]

Figure S1

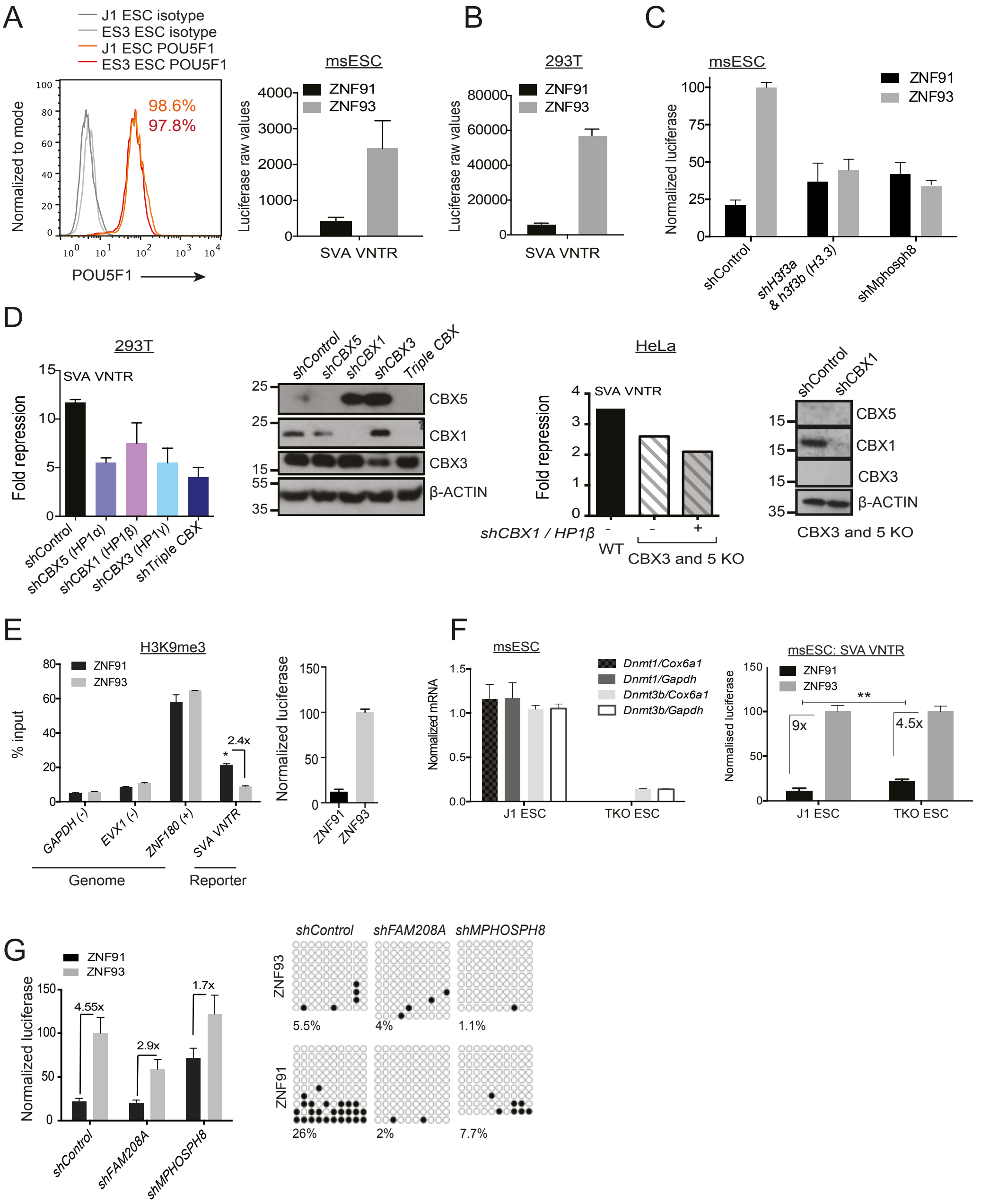
A
J1 ESC isotype
ES3 ESC isotype
J1 ESC POU5F1
ES3 ESC POU5F1
Normalized to mode
98.6%
97.8%
POU5F1
msESC
Luciferase raw values
ZNF91
ZNF93
SVA VNTR
B
293T
C
msESC
Normalized luciferase
shControl
shH3f3a & h3f3b (H3.3)
shMphosph8
D
293T
SVA VNTR
Fold repression
shControl
shCBX5 (HP1α)
shCBX1 (HP1β)
shCBX3 (HP1γ)
shTriple CBX
shCBX5
shCBX1
shCBX3
Triple CBX
CBX5
CBX1
CBX3
β-ACTIN
HeLa
shCBX1 / HP1β
WT
CBX3 and 5 KO
E
H3K9me3
% input
GAPDH (-)
EVX1 (-)
ZNF180 (+)
Genome
Reporter
2.4x
F
msESC
Dnmt1/Cox6a1
Dnmt1/Gapdh
Dnmt3b/Cox6a1
Dnmt3b/Gapdh
Normalized mRNA
J1 ESC
TKO ESC
msESC: SVA VNTR
Normalised luciferase
9x
4.5x
G
4.55x
2.9x
1.7x
shFAM208A
shMPHOSPH8
shMphosph8
5.5%
4%
1.1%
26%
2%
7.7%

Supplemental Fig S1.

(A) Two genetic strains of msESCs (J1 and ES3) were verified to express POU5F1 (left) and subject to reporter assays (right, ES3 shown). Raw luciferase values are given to show the reporter assay is robust in msESCs. (B) Despite the reporter containing a *POU5F1* enhancer, luciferase expression does not depend on POU5F1 as it is robust in POU5F1-negative 293T cells. (C) Reporter assays in ES3 msESCs depleted for H3F3A/B (H3.3) or MPHOSPH8. Results are normalized to the shControl + ZNF93. (D) Reporter assays were performed in 293T cells depleted for CBX family members (left) or HeLa cells double knockout (CRISPR/Cas9-mediated) for CBX5 and CBX3 (HP1alpha and gamma) expressing a hairpin for CBX1 (HP1beta) (right). The greatest relief of repression was upon triple CBX (HP1) depletion. Western blots show depletion efficiencies. (E) H3K9me3 ChIP on 293T cells 48h post-reporter transfection. Results show IPs normalized to TIs. H3K9me3 enrichment was measured at the reporter (SVA VNTR) and in the genome. Rabbit polyclonal IgG was used as a negative control (giving no enrichment, not shown). Genomic *GAPDH* and *EVX1* were internal negative control regions and *ZNF180* was a positive control. One representative experiment of two is shown and error bars show standard error (sem) of technical replicates. A two-tailed paired t test was performed on both experiments showing significant H3K9me3 accumulation on the reporter ($p=0.029$). (F) Triple knockout (TKO) msESCs that are *Dnmt1-/-, Dnmt3a-/-* and *Dnmt3b-/-* (from Masaki Okano) were verified to be depleted of DNMT1 and DNMT3B by qRT-PCR using their parent cell line, J1 ESCs as a control (left panel). J1 ESCs and TKO cells were co-transfected in triplicate with reporter assays (right panel). Repression was significantly enhanced in control compared to TKO cells (unpaired t-test, $p=0.0042$). (G) Relates to Figure 1G. Left: reporter assay results done in the same cells that were subject to DNA methylation analysis that is shown in Figure 1G. Results were all normalized to the shControl + ZNF93 bar.

Fold repression between ZNF91 and the control ZNF93 are given. Right: raw DNA methylation data from one representative experiment for which summary data are shown in Figure 1G.

Figure S2

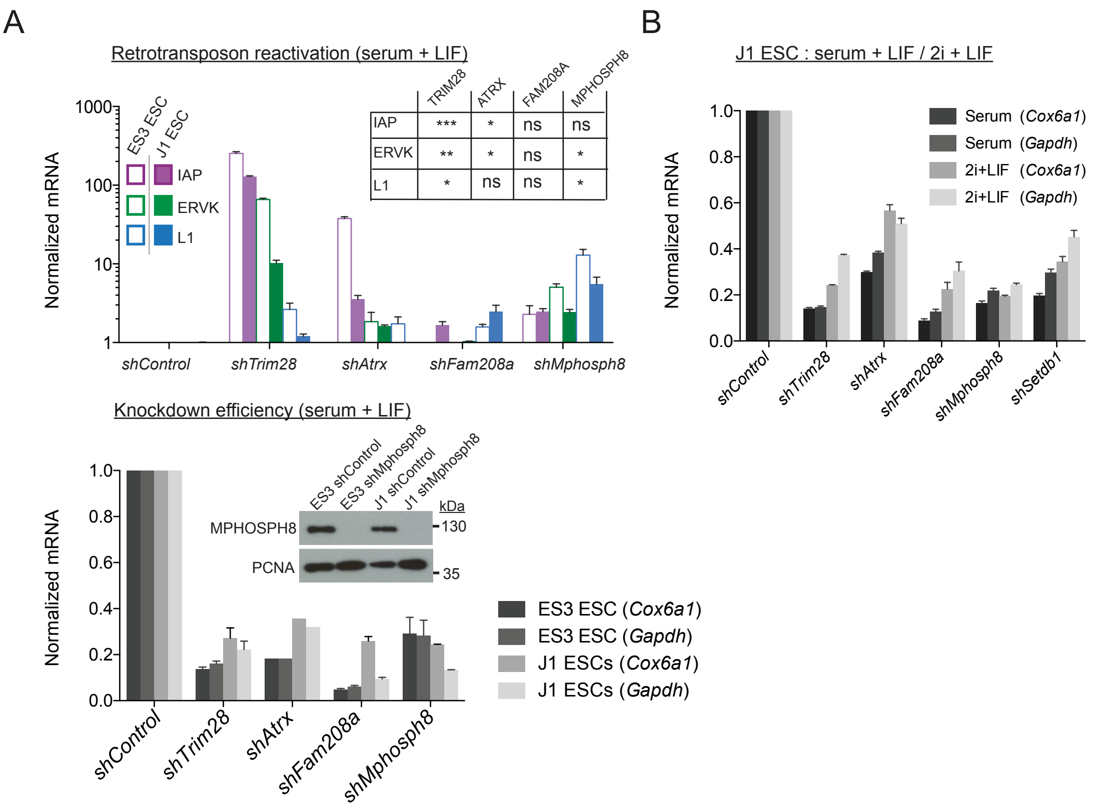

A
Retrotransposon reactivation (serum + LIF)
Normalized mRNA
ES3 ESC
J1 ESC
IAP
ERVK
L1
TRIM28
ATRX
FAM208A
MPHOSPH8
IAP *** * ns ns
ERVK ** * ns *
L1 * ns ns *
shControl
shTrim28
shAtrx
shFam208a
shMphosph8
Knockdown efficiency (serum + LIF)
ES3 shControl
ES3 shMphosph8
J1 shControl
J1 shMphosph8
kDa
MPHOSPH8
130
PCNA
35
ES3 ESC (Cox6a1)
ES3 ESC (Gapdh)
J1 ESCs (Cox6a1)
J1 ESCs (Gapdh)
B
J1 ESC : serum + LIF / 2i + LIF
Serum (Cox6a1)
Serum (Gapdh)
2i+LIF (Cox6a1)
2i+LIF (Gapdh)
shSetdb1

Supplemental Fig S2.

(A) ES3 and J1 msESCs cultured in serum + LIF were depleted for epigenetic factors and expression of retrotransposons measured by qRT-PCR. Data shown are from one representative experiment of two, normalized to *Gapdh*. The table shows p values (two-tailed paired t tests) for this and repeat experiments (N=2 for ES3 ESCs and N=2 for J1 ESCs). Knockdown efficiency was verified by qRT-PCR and Western blot for MPHOSPH8 (bottom) and TRIM28 (not shown). Expected sizes, MPHOSPH8: 97kDa. (B) Depletion of epigenetic factors was verified by qRT-PCR for J1 ESCs cultured in serum + LIF vs. 2i + LIF, which relates to the experiment depicted in Figure 3C. Data were normalized to *Cox6a1* and *Gapdh*.

Figure S3

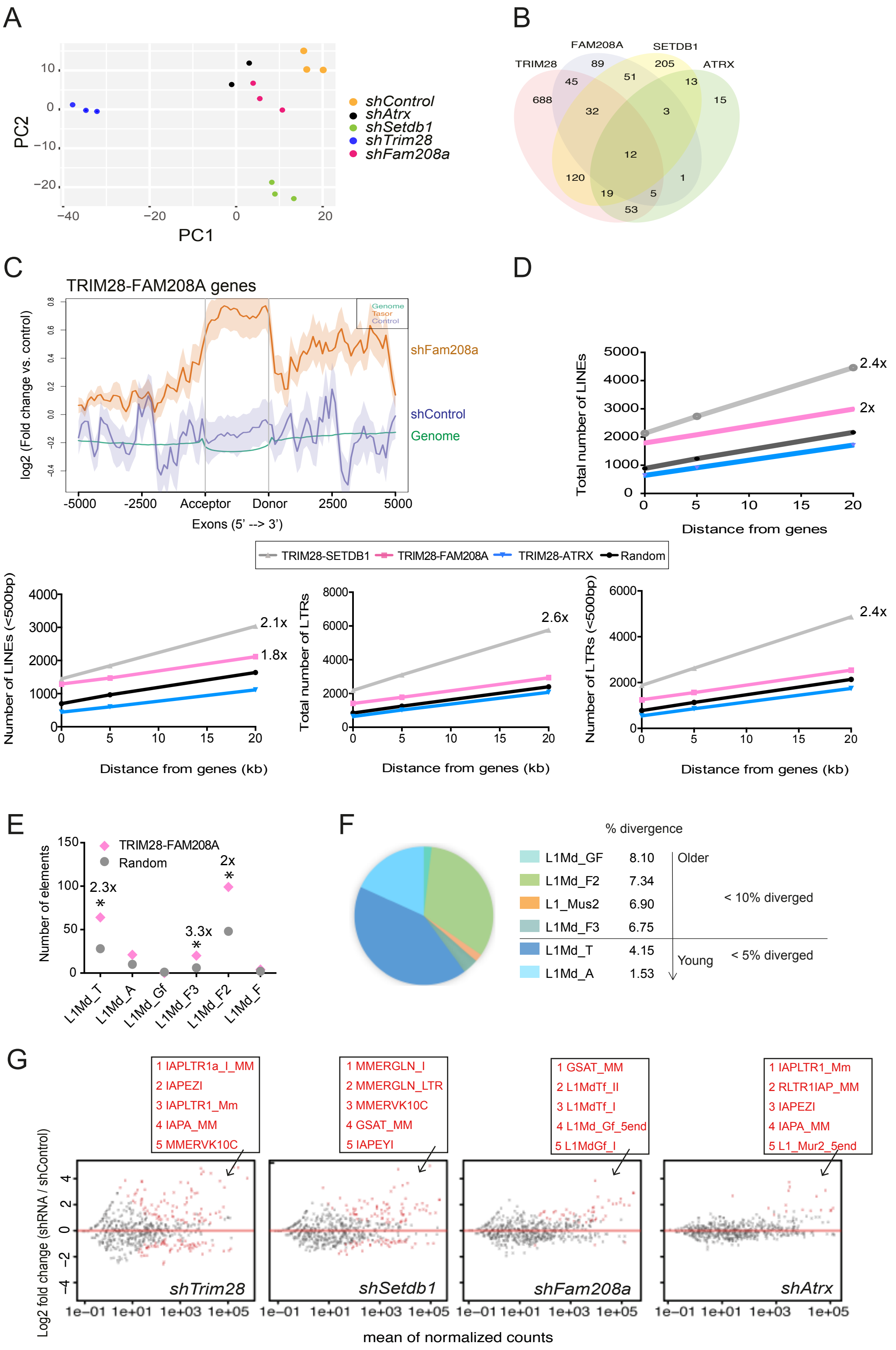
A
PC2
PC1
shControl
shAtrx
shSetdb1
shTrim28
shFam208a
B
TRIM28
FAM208A
SETDB1
ATRX
C
TRIM28-FAM208A genes
log2 (Fold change vs. control)
shFam208a
shControl
Genome
Acceptor
Donor
Exons (5' --> 3')
D
Total number of LINEs
Distance from genes
TRIM28-SETDB1
TRIM28-FAM208A
TRIM28-ATRX
Random
Number of LINEs (<500bp)
Total number of LTRs
Number of LTRs (<500bp)
Distance from genes (kb)
E
Number of elements
TRIM28-FAM208A
Random
L1Md_T
L1Md_A
L1Md_Gf
L1Md_F3
L1Md_F2
L1Md_F
F
% divergence
L1Md_GF 8.10
L1Md_F2 7.34
L1_Mus2 6.90
L1Md_F3 6.75
L1Md_T 4.15
L1Md_A 1.53
Older
< 10% diverged
Young
< 5% diverged
G
1 IAPLTR1a_I_MM
2 IAPEZI
3 IAPLTR1_Mm
4 IAPA_MM
5 MMERVK10C
1 MMERGLN_I
2 MMERGLN_LTR
3 MMERVK10C
4 GSAT_MM
5 IAPEYI
1 GSAT_MM
2 L1MdTf_II
3 L1MdTf_I
4 L1Md_Gf_5end
5 L1MdGf_I
1 IAPLTR1_Mm
2 RLTR1IAP_MM
3 IAPEZI
4 IAPA_MM
5 L1_Mur2_5end
Log2 fold change (shRNA / shControl)
shTrim28
shSetdb1
shFam208a
shAtrx
mean of normalized counts

Supplemental Fig S3.

(A) Principal component analysis (PCA) shows that treatment groups cluster together. Each group has 3 samples except ATRX, which has 2. (B) Venn diagram of all the upregulated genes between the different mRNA-seq treatment groups. (C) Ngs.plots that show coverage of reads across the exons of the 94 TRIM28-FAM208A genes (depicted between the two vertical lines) in the *shControl* vs. the *shFam208a* samples vs. the genome. The whole length of transcripts are upregulated, although coverage is not completely even because otherwise it would be flat. (D) The rest of the results from Figure 3F are shown here. Fold changes relative to random genes are stated where differences were observed. (E) In a complementary analysis to (D) where L1s were assessed by length, here we assessed them by type independent of length. We used the UCSC table browser to intersect gene groups with bed files of stated L1 families. TRIM28-FAM208A genes were significantly enriched for the TRIM28-regulated L1s, L1Md_T, L1Md_F3 and L1Md_F2 within them compared to random genes. (F) The same identified L1 integrants in Figure 3H were re-classified here by age this time based on their mean divergence from consensus sequences. All integrants were less than 10% diverged. (G) MA plots showing differences in retrotransposon expression between each treatment group and the shControl. Repeats that exhibit a significant (where adjusted p values =<0.01) up or downregulation are displayed in red. The top 5 upregulated repeat names are annotated in each top right corner box.

Figure S4

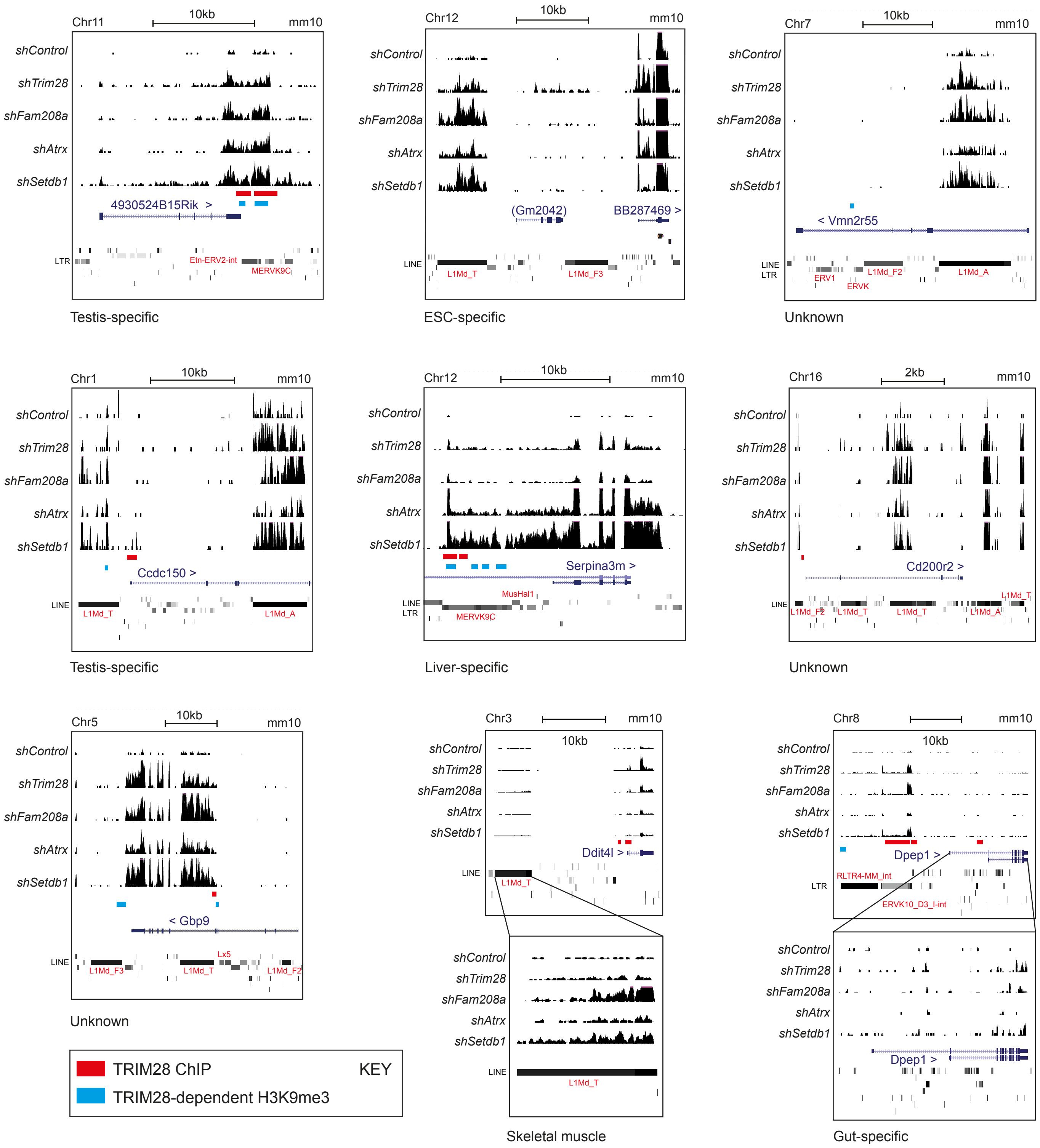

Chr11
10kb
mm10
shControl
shTrim28
shFam208a
shAtrx
shSetdb1
4930524B15Rik >
LTR
Etn-ERV2-int
MERVK9C
Testis-specific
Chr12
10kb
mm10
(Gm2042)
BB287469 >
LINE
L1Md_T
L1Md_F3
ESC-specific
Chr7
10kb
mm10
< Vmn2r55
LINE
LTR
ERV1
ERVK
L1Md_F2
L1Md_A
Unknown
Chr1
10kb
mm10
Ccdc150 >
LINE
L1Md_T
L1Md_A
Testis-specific
Chr12
10kb
mm10
Serpina3m >
LINE
LTR
MusHal1
MERVK9C
Liver-specific
Chr16
2kb
mm10
Cd200r2 >
LINE
L1Md_F2
L1Md_T
L1Md_T
L1Md_T
L1Md_A
Unknown
Chr5
10kb
mm10
< Gbp9
LINE
Lx5
L1Md_F3
L1Md_T
L1Md_F2
Unknown
Chr3
10kb
mm10
Ddit4l >
LINE
L1Md_T
L1Md_T
Skeletal muscle
Chr8
10kb
mm10
Dpep1 >
RLTR4-MM_int
LTR
ERVK10_D3_I-int
Dpep1 >
Gut-specific
TRIM28 ChIP
TRIM28-dependent H3K9me3
KEY

Supplemental Fig S4.

mRNA-sequencing tracks (see methods) of naive J1 msESC depleted of the stated epigenetic factors. TRIM28 binding (Castro-Diaz et al. 2014) and TRIM28-dependent H3K9me3 (Rowe et al. 2013b) are annotated. TRIM28-dependent H3K9me3 was previously defined as H3K9me3 peaks that are lost in TRIM28-depleted msESCs (Rowe et al. 2013b).

Figure S5

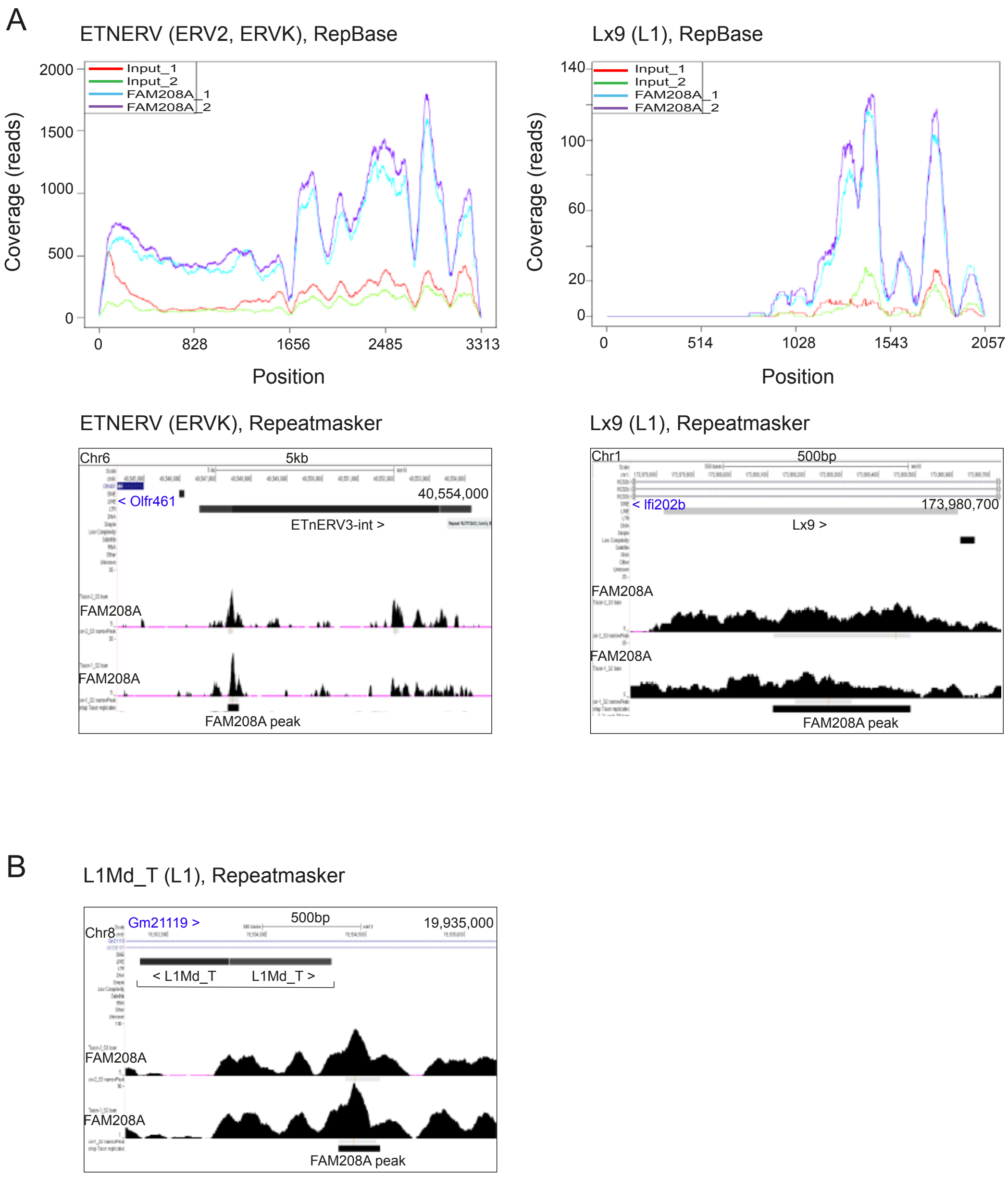
A
ETNERV (ERV2, ERVK), RepBase
Input_1
Input_2
FAM208A_1
FAM208A_2
Coverage (reads)
Position
Lx9 (L1), RepBase
ETNERV (ERVK), Repeatmasker
Chr6
5kb
< Olfr461
40,554,000
ETnERV3-int >
FAM208A
FAM208A peak
Lx9 (L1), Repeatmasker
Chr1
500bp
< Ifi202b
173,980,700
Lx9 >
FAM208A
FAM208A peak
B
L1Md_T (L1), Repeatmasker
Chr8
Gm21119 >
500bp
19,935,000
< L1Md_T
L1Md_T >
FAM208A
FAM208A peak

Supplemental Fig S5.

(A) Example ERV and L1 bound by FAM208A. Upper panels show reads mapped directly to the stated RepBase consensus sequences. FAM208A IP samples are shown in blue and purple. Lower panels show UCSC screenshots of FAM208A binding at the same retrotransposon families but this time following mapping of reads to the genome. Tracks show bigWig files and the FAM208A peak identified underneath as determined by MACS2. (B) UCSC screenshot of FAM208A binding at a young L1Md_T element in the genome. Note that we could not detect any specific binding of FAM208A to young L1 RepBase consensus sequences (including L1Md_T) compared to total input samples (not shown).

Figure S6

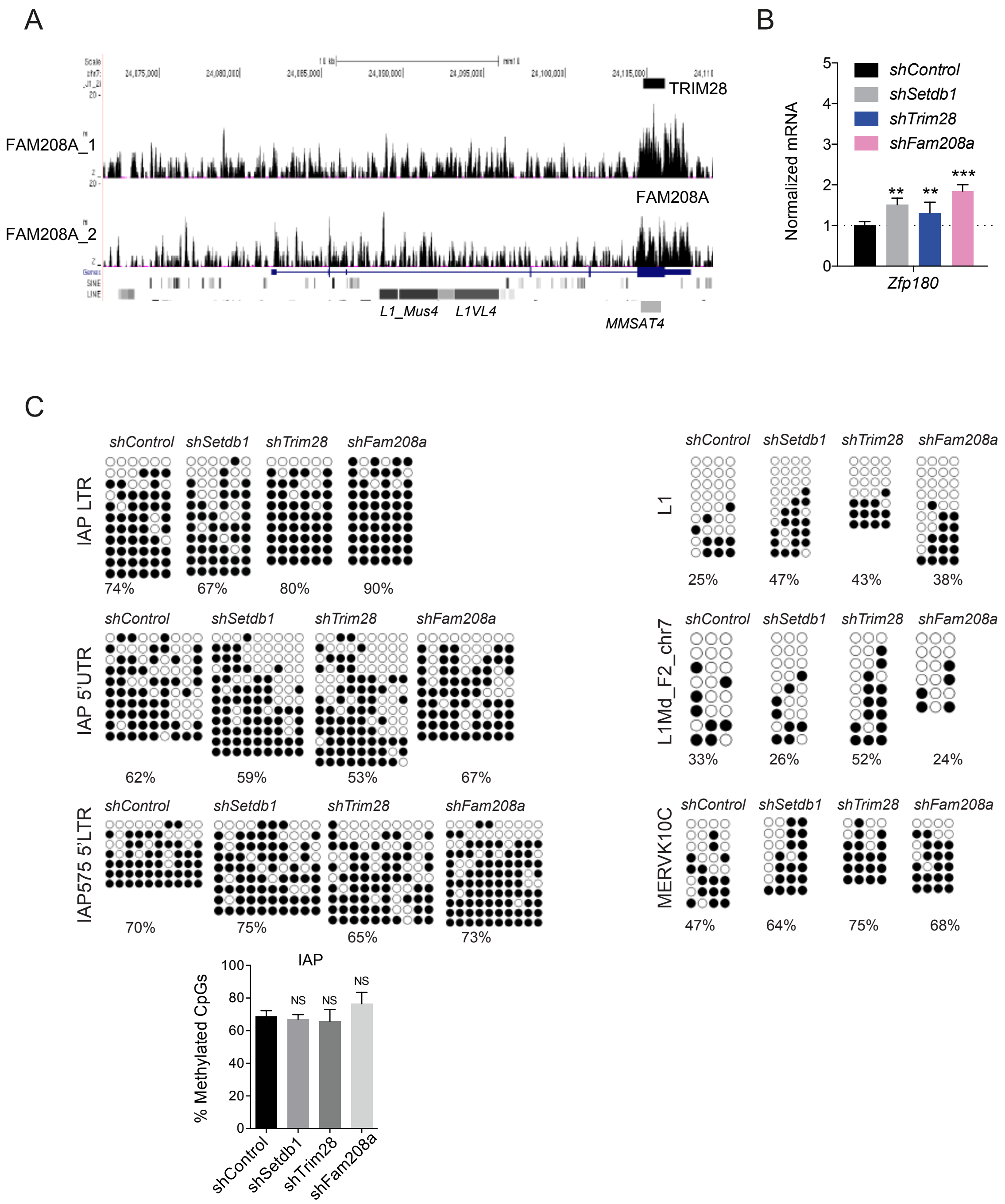
A
TRIM28
FAM208A_1
FAM208A
FAM208A_2
L1_Mus4
L1VL4
MMSAT4
B
shControl
shSetdb1
shTrim28
shFam208a
Normalized mRNA
Zfp180
C
IAP LTR
74%
67%
80%
90%
IAP 5'UTR
62%
59%
53%
67%
IAP575 5'LTR
70%
75%
65%
73%
L1
25%
47%
43%
38%
L1Md_F2_chr7
33%
26%
52%
24%
MERVK10C
47%
64%
75%
68%
IAP
% Methylated CpGs
NS

Supplemental Fig S6.

(A) UCSC map of the mouse *Zfp180* locus showing TRIM28 and FAM208A co-binding. TRIM28 data are mapped peaks from (Castro-Diaz et al. 2014) and FAM208A data are bigWig files from this study. (B) qRT-PCR results showing *Zfp180* expression. A summary of 3 experiments is shown with two-tailed unpaired t-tests. (C) DNA methylation analysis at endogenous IAP, MERVK10C and L1 retrotransposons 4 days after depletion of epigenetic factors in J1 ESCs (2i+LIF cultured). Statistical results are representative of the three independent depicted measurements made on DNA methylation levels at IAP elements. Note that IAP LTR and L1 shControl data are also shown in Figure 6D.

Figure S7

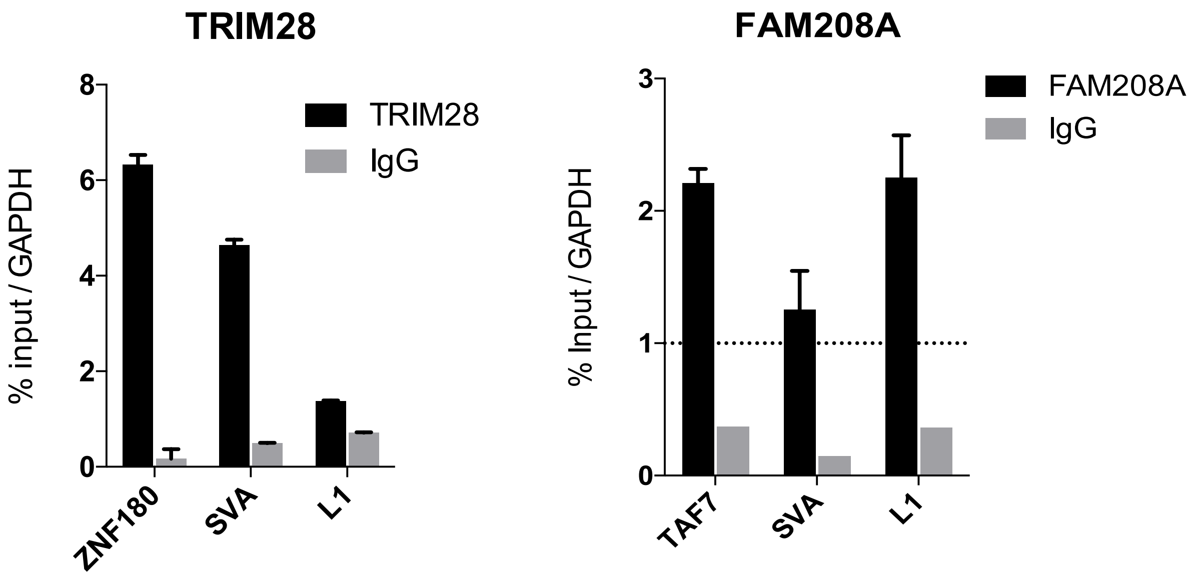
TRIM28
8
6
4
2
0
% input / GAPDH
TRIM28
IgG
ZNF180
SVA
L1
FAM208A
3
2
1
0
% Input / GAPDH
FAM208A
IgG
TAF7
SVA
L1

Supplemental Fig S7.

The same ChIP-PCR results from Figure 6J are illustrated here showing enrichment of TRIM28 (left) and FAM208A (right) on SVA and L1 elements but this time with the respective IgG controls shown. ZNF180 was a positive control for TRIM28 enrichment and TAF7 was a positive control for FAM208A enrichment.
